# A comprehensive analysis of cardiovascular mortality trends in Peru from 2017 to 2022: Insights from 183,386 deaths of the national death registry

**DOI:** 10.1016/j.ahjo.2023.100335

**Published:** 2023-10-20

**Authors:** Hugo G. Quezada-Pinedo, Noushin Sadat Ahanchi, Kim N. Cajachagua-Torres, Jordan A. Obeso-Manrique, Luis Huicho, Christoph Gräni, Taulant Muka

**Affiliations:** a The Generation R Study Group, Erasmus MC, University Medical Center Rotterdam, Rotterdam, the Netherlands; b Department of Cardiology, Bern University Hospital, University of Bern, Bern, Switzerland; c Centro de Investigación en Salud Materna e Infantil and Centro de Investigación para el Desarrollo Integral y Sostenible, Universidad Peruana Cayetano Heredia, Lima, Peru; d Institute of Social and Preventive Medicine (ISPM), Graduate School of Health Sciences, University of Bern, Bern, Switzerland; e Department of Pediatrics, New York University Grossman of Medicine, New York, USA; f Epistudia, Bern, Switzerland; g Facultad de Medicina “Alberto Hurtado”, Universidad Peruana Cayetano Heredia, Lima, Peru; h Graduate School for Health Sciences, University of Bern, Bern, Switzerland; i Department of Internal Medicine, Internal Medicine, Lausanne University Hospital, Lausanne, Switzerland

**Keywords:** Cardiac mortality, Cardiovascular health, Low- and middle-income countries, Death registry, Peru

## Abstract

**Background/objectives:**

Cardiovascular diseases are the leading cause of global mortality. Systematic studies on cardiovascular-related mortality at national and subnational levels in Peru are lacking. We aimed to describe the trends in cardiovascular-related mortality between 2017 and 2022 in Peru at national and subnational levels and by socioeconomic indicators.

**Subjects/methods:**

We used data from the Peruvian death registry 2017–2022. Using ICD-10 codes, mortality was categorized into: hypertensive-, coronary-, and cerebrovascular- related deaths. We estimated age-standardized cardiovascular-related mortality rates by sex at national and regional levels, and by natural regions (Coast, Highlands, Amazon). We estimated the change in mortality rates between 2017–2019 and 2020–2022 and explored factors that contributed to such a change. We explored ecological relationships between mortality rates and socioeconomic indicators.

**Findings:**

Overall 183,386 cardiovascular-related deaths were identified. Coronary-related deaths (37.2 %) were followed by hypertensive-related (25.1 %) and cerebrovascular-related deaths (22.6 %). Peru showed a marked increasing trend in cardiovascular-related mortality in 2020–2022 (77.8 %). The increase clustered in the Coast and Highlands, with the highest change observed in Lima (132.1 %). Mortality was highest in subjects with lower education and subjects with public health insurance. Gini coefficient was associated with lower mortality rates while unemployment was associated with higher mortality rates.

**Interpretation:**

There was a notable rise in cardiovascular-related mortality in Peru, particularly during the Covid-19 pandemic with a slight decrease in 2022. Gaining a comprehensive understanding of the factors that contribute to the increase in cardiovascular deaths in Peru will facilitate the development of precise interventions at both the national and regional levels.

**Research in context**

**Evidence before this study**

On 24 April 2023 a search without date and language restrictions was conducted in Pubmed using the following search terms: “cardiovascular mortality AND Latin America countries”. From a total of 167 articles found, 6 studies evaluating national trends in cardiovascular-related mortality were identified. All studies evaluated cardiovascular-related mortality at country-level as part of a global or regional study and all were conducted before the onset of the Covid-19 pandemic in 2020. These studies did not evaluate subnational trends that would allow to disentangle differences between regions within a country.

**Added value of this study**

To the best of our knowledge, this study is unique in evaluating age-standardized cardiovascular-related mortality rates by cardiovascular phenotypes including hypertensive-, coronary- and cerebrovascular-related deaths at a national and subnational level in Peru for a recent period of time. The study provides an in-depth evaluation at national, regional and regional levels, while also including an analysis by socioeconomic status. An increased trend in cardiovascular-related mortality rates with a notable increase since 2020 was observed at all studied levels. Deaths were clustered in the Coast of Peru and were influenced by Gini coefficient and unemployment.

**Implications of all the available evidence**

The increased trend of age-standardized cardiovascular-related mortality rates at all levels, with a dramatic increase since 2020, advocate for an exhaustive analysis to identify the drivers of the high rates at different regional levels. This includes the evaluation of the determinants of health at different levels, considering factors inside and outside the health sector and the review of the current health interventions and guidelines. National and local authorities should be aware of the increased rate of cardiovascular-related mortality and of the urgency of implementing locally-adapted policies to tackle effectively the problem.

Alt-text: Unlabelled box

## 1 Introduction

Cardiovascular diseases (CVDs) are collectively one of the main global causes of concern due to the rising prevalence, the resulting mortality and disability, along with heavy financial burden worldwide [1]. Prevalent cases of total CVD nearly doubled from 271 million in 1990 to 523 million in 2019, and the number of CVD deaths steadily increased from 12.1 million in 1990 to 18.6 million in 2019, representing a 65 % increase in this period [2]. However, the mortality rates vary between countries. In high-income countries, the substantial decline in CVDs mortality over the past half-century has been a major global public health achievement [3,4]. On the other hand, >75 % of CVD fatalities can be attributed to low- and middle-income countries (LMICs), which have also experienced a notable rise in the prevalence of CVDs in recent years [5].

Latin America is facing an epidemiologic transition from infectious to chronic diseases and is likely to face a larger epidemic of heart disease in the next years unless urgent action is taken [6]. It is growingly being recognized that the mortality of CVDs and their contributing determinants may differ between and within countries due to different levels of socioeconomic development and heterogeneity in health systems [7]. Therefore in-depth country level analyses of CVDs mortality are largely warranted [7,8].

Given the elevated occurrence of cardiovascular risk factors in Peru, it becomes increasingly crucial to revise and update mortality studies. Results of a multicenter cohort indicated a low prevalence of ideal cardiovascular health metrics in Peruvian adults [7]. Moreover, a nationally representative survey reported that the prevalence of hypertension, diabetes, and obesity was 9.8 %, 4.9 %, and 25.8 %, respectively, in 2021 [9]. Previous studies in other countries showed that the Covid-19 pandemic has resulted in an inflation of acute cardiovascular deaths [[10], [11], [12]]. Despite the high prevalence of cardiovascular risk factors in Peru and evidence suggesting an increase in cardiovascular-related mortality in the region, there is a lack of systematic assessments of cardiovascular-related mortality trends.

Thus, we aimed to describe national and subnational trends of cardiovascular-related mortality in Peru between 2017 and 2022 in relation to time, geographical location and socioeconomic factors. This study will help to identify areas at higher risk and guide the central and local decision makers in the design and implementation of health interventions.

## 2 Methods

### 2.1 Data sources

The Peruvian national death register, SINADEF (Sistema Informático Nacional de Defunciones, in Spanish) was used as the source of deaths data. The registry was implemented in 2017 and contains individualized information from all the deaths occurring across the Peruvian territory [13]. The registry also included socioeconomic information including education and health insurance. Data can be accessed directly from the Peruvian open data web page: https://www.datosabiertos.gob.pe/dataset/sinadef-certificado-defunciones. The coverage of cause-of-deaths in the SINADEF register, calculated as the ratio of the total number of deaths that have been registered with cause-of-death information in the vital registration system for a country-year to the total estimated deaths for that year, was estimated to be 70 % for the years 2017, 2018, and 2019 [14,53]. However, there is no available information on the coverage for the period between 2020 and 2022. We additionally collected available information from the National Institute of Statistics and Computing, INEI (Instituto Nacional de Estadística e Informatica, in Spanish) on gross domestic product (GDP) per capita in USD, Gini coefficient for income, percentage of urban population, percentage of poverty, average per capita monthly income in USD, unemployment rate, median years of schooling, percentage of population with health insurance, and cardiovascular-related outpatient visits rate [15].

### 2.2 Study setting

Located in South America, Peru is classified as an upper-middle income country according to the World Bank classification [16]. Nonetheless, economic disparities between regions still persist (Supplementary Table 1) [13]. In 2022, Peru had 33,396,700 inhabitants [17] and its gross domestic product for 2021 was 223.3 billion US dollars [18]. The country is divided in three natural regions: Coast, Highland and the Amazon [17]. It is politically composed of 24 departments that are further divided in 196 provinces [17]. These 24 regions could be grouped in one of the three natural regions. The Coast corresponds to the areas close to the Pacific Ocean, the Highlands to the mountainous Andean region and the Amazon to the rainforest areas [17]. Peru has three main healthcare providers, namely the comprehensive health insurance SIS (Seguro Integral de Salud, in Spanish) administered by the Ministry of Health and covering 64 % of the population, the Social Insurance ESSALUD (Seguro Social de Salud, in Spanish) administered by the Ministry of Labour and covering 29 % of the population, and the private sector [19].

### 2.3 Study population

All deaths from the national death register between 2017 and 2022 were included for this study. We conducted a complete case analysis and excluded participants with missing values for age, cause of death or location of death (Supplementary Fig. 1). We observed that Lambayeque exhibited inconsistent numbers of deaths throughout the study period, with the following number of deaths for each year: 2017 (*n* = 1912), 2018 (*n* = 1235), 2019 (*n* = 90), 2020 (*n* = 2081), 2021 (*n* = 740), and 2022 (*n* = 1346). We do not know the reason for this discrepancy but the problem could be related with underreporting. For this reason, we decided to excluded Lambayeque from all our analyses.

### 2.4 Definitions

Age-standardized cardiovascular-related mortality rate was estimated according to the direct method using population age structures by region per 5-years increments and population projections provided by the National Institute of Statistics and Computing [20,21]. Thus, age-standardized mortality rate was calculated by first estimating age-specific mortality rates for each age group. This was achieved by dividing the number of deaths within each age group by the corresponding population [22]. Then, each age-specific rates were multiplied by the standard weight belonging to the particular age group. Finally, the age-standardized rate is obtained by adding the resulting numbers [22].

The immediate cause of death “a” as well as the antecedent causes of death “b”, “c” and “d” were counted as a case and used to calculate the numerator of death by cardiovascular-related causes. This approach was used because antecedent causes of death are considered part of the main causal sequence leading to death and directly contributing to the immediate cause of death [23,24]. Based on the registered ICD-10 codes, cardiovascular mortality was categorized into the following classification: cardiovascular disease (I00-I99), hypertensive disease (I10-I15), coronary disease (I20-I25), and cerebrovascular disease (I60-I69). Cardiovascular-related mortality was described in relation to time, regional and geographical levels, phenotypes and sex and in relation to socioeconomic characteristics, namely education (none/any elementary school/incomplete high school, complete high school and any higher than high school), health insurance provider (ESSALUD, out-of-pocket health expenditure, private and SIS).

### 2.5 Statistical analysis

Age-standardized cardiovascular mortality rates were expressed per 100,000 and estimated by sex and region [20]. Additionally, we calculated the standard error (SE) with the following formula: $\mathrm{SE}=\frac{\mathrm{standard mortality rates}}{\sqrt{\mathrm{number of deaths}}}$ and then we calculated the 95 % confidence intervals (95%CI) as follows: standard mortality rates ±1.96*SE [25]. To show spatial and temporal patterns, maps and time trends were elaborated. Equiplots were developed to show differences in cardiovascular-related mortality based on education and health insurance. To explore changes in cardiovascular-related mortality during the study period we estimated the excess death rate as the difference between the average of the periods 2017–2019 and 2020–2022. We selected these two periods because the COVID-19 pandemic started in 2020. Ecological associations at regional level between annual cardiovascular-related mortality rates and selected socioeconomic variables (i.e., gross domestic product (GDP) per capita, Gini coefficient for income, percentage of urban population, percentage of poverty, average per capita monthly income (USD), unemployment rate, median years of schooling, percentage of people with health insurance and cardiovascular-related outpatients visit rate) were evaluated using linear mixed-effects models. This approach has advantages in data with missing values and a hierarchical structure and it allows to control for the variability between and within regions [13,[26], [27], [28], [29]]. For this, all predictor predictors were simultaneously included in the model, along with time as a dummy variable indicating observation collected before (=0) or after (=1) the beginning of the Covid-19 pandemic to explore the effects of the pandemic [13,[26], [27], [28], [29]]. We employed linear mixed-effects regressions as a hypothesis-generating approach to aid in the exploration of potential associations. It's important to note that these results are susceptible to ecological fallacy and should not be interpreted as implying causal relationships at individual level [13,[26], [27], [28], [29]]. R statistical software version 4.2.1 (R foundation, Vienna, Austria) was used for all the analyses. The study is reported according to the Strengthening the Reporting of Observational Studies in Epidemiology (STROBE) guidelines.

### 2.6 Ethics

The data used in this project was retrieved from open-source websites. The study was approved by the Research Ethics Committee of the Universidad Peruana Cayetano Heredia (UPCH), Lima, Peru (CIEI 312–29-23).

## 3 Results

### 3.1 Study population characteristics

From the initial 190,806 cardiovascular-related deaths, we excluded 7404 deaths from the Lambayeque region due to inconsistent reporting across years. Among the remined 183,402 cardiovascular-related deaths available in the death registry, we further excluded 16 deaths due to missing values in either age of death or place of death. The final sample size was 183,386 deaths, of which 68,269 correspond to coronary deaths, 45,991 to hypertensive deaths, deaths and 41,380 to cerebrovascular deaths. Out of the total, 48.6 % of deaths were females, the median (25th percentile, 75th percentile) age of death of the study population was 78.0 (65.0, 86.0) years, 46.5 % of them were married, 12.5 % had any higher than high school education and 62.3 % were users of SIS health insurance (Table 1).

**Table 1:** Characteristics of the study population by year.

Year	2017 *n* = 18,521	2018 *n* = 20,363	2019 *n* = 21,934	2020 *n* = 40,549	2021 *n* = 45,756	2022 *n* = 36,263
Sex, females, n (%)	9047 (48.9)	10,011 (49.2)	10,822 (49.3)	18,917 (46.7)	22,189 (48.5)	18,136 (50.0)
Age, years, median (25th–75th percentile)	64.0 (77.0, 86.0)	64.0 (77.0, 86.0)	65.0 (78.0, 87.0)	66.0 (78.0, 86.0)	65.0 (78.0, 86.0)	66.0 (79.0, 87.0)
Civil status, married, n (%)	7′690 (43.6)	8′946 (45.3)	9909 (46.2)	19,377 (48.6)	21,628 (47.7)	16′169 (45.2)
Education, n (%)						
None/any elementary school/incomplete high school	11,360 (72.4)	12,282 (71.9)	12,757 (69.7)	22,613 (66.4)	24,524 (65.2)	19,555 (67.9)
Complete high school	2692 (17.1)	2816 (16.5)	3187 (17.4)	7007 (20.6)	8126 (21.6)	5670 (19.7)
Any higher than high school	1645 (10.5)	1981 (11.6)	2365 (12.9)	4432 (13.0)	4944 (13.2)	4079 (13.1)
Health insurance, n (%)						
SIS^a^	8085 (66.6)	10,226 (64.6)	11,610 (61.7)	20,425 (58.7)	25,454 (61.8)	21,666 (64.4)
ESSALUD^b^	3193 (26.3)	4829 (30.5)	6395 (34.0)	12,968 (37.2)	14,311 (34.7)	10,867 (32.3)
Private/out-of-pocket health expenditure/other	1434 (11.3)	1497 (9.0)	1550 (7.9)	2790 (7.7)	2687 (6.3)	1794 (5.2)
Deaths by age categories, n (%)						
0–5	123 (0.7)	98 (0.5)	89 (0.4)	135 (0.3)	127 (0.3)	133 (0.4)
6–10	91 (0.5)	83 (0.4)	72 (0.3)	97 (0.2)	112 (0.2)	101 (0.3)
11–14	92 (0.5)	74 (0.4)	71 (0.3)	105 (0.3)	120 (0.3)	144 (0.4)
15–19	120 (0.6)	112 (0.6)	135 (0.6)	147 (0.4)	193 (0.4)	178 (0.5)
20–24	173 (0.9)	194 (1.0)	153 (0.7)	229 (0.6)	243 (0.5)	246 (0.7)
25–29	227 (1.2)	210 (1.0)	201 (0.9)	314 (0.8)	335 (0.7)	309 (0.9)
30–34	214 (1.2)	231 (1.1)	228 (1.0)	354 (0.9)	471 (1.0)	365 (1.0)
35–39	264 (1.4)	269 (1.3)	285 (1.3)	524 (1.3)	602 (1.3)	489 (1.3)
40–44	391 (2.1)	428 (2.1)	432 (2.0)	739 (1.8)	871 (1.9)	648 (1.8)
45–49	500 (2.7)	530 (2.6)	567 (2.6)	1002 (2.5)	1220 (2.7)	862 (2.4)
50–54	728 (3.9)	754 (3.7)	704 (3.2)	1325 (3.3)	1724 (3.8)	1179 (3.3)
55–59	871 (4.7)	954 (4.7)	969 (4.4)	1850 (4.6)	2299 (5.0)	1619 (4.5)
60–64	1107 (6.0)	1246 (6.1)	1304 (5.9)	2565 (6.3)	2988 (6.5)	2037 (5.6)
65–69	1357 (7.3)	1543 (7.6)	1626 (7.4)	3344 (8.2)	3715 (8.1)	2687 (7.4)
70–74	1859 (10.0)	2092 (10.3)	2222 (10.1)	4076 (10.1)	4691 (10.3)	3343 (9.2)
75–79	2262 (12.2)	2524 (12.4)	2663 (12.1)	5053 (12.5)	5471 (12.0)	4328 (11.9)
>80	8142 (44.0)	9021 (44.3)	10,213 (46.6)	18,690 (46.1)	20,574 (45.0)	17,595 (48.5)
Deaths by ICD-10, n (%)						
I00-I99 (cardiovascular diseases)	18,521	20,363	21,934	40,549	45,756	36,263
I10-I15 (hypertensive diseases)	4082 (22.04)	4568 (22.43)	4921 (22.44)	11,300 (27.87)	12,284 (26.85)	8836 (24.37)
I20-I25 (coronary heart disease)	4813 (25.99)	5519 (27.1)	6345 (28.93)	16,655 (41.07)	19,823 (43.32)	15,114 (41.68)
I60-I69 (cerebrovascular disease)	4735 (25.57)	5690 (27.94)	6013 (27.41)	8355 (20.6)	8974 (19.61)	7613 (20.99)

a SIS: Seguro Integral de Salud in Spanish.

b ESSALUD: Seguro Social de Salud in Spanish.

### 3.2 Geographical trends

**Fig. 1:**
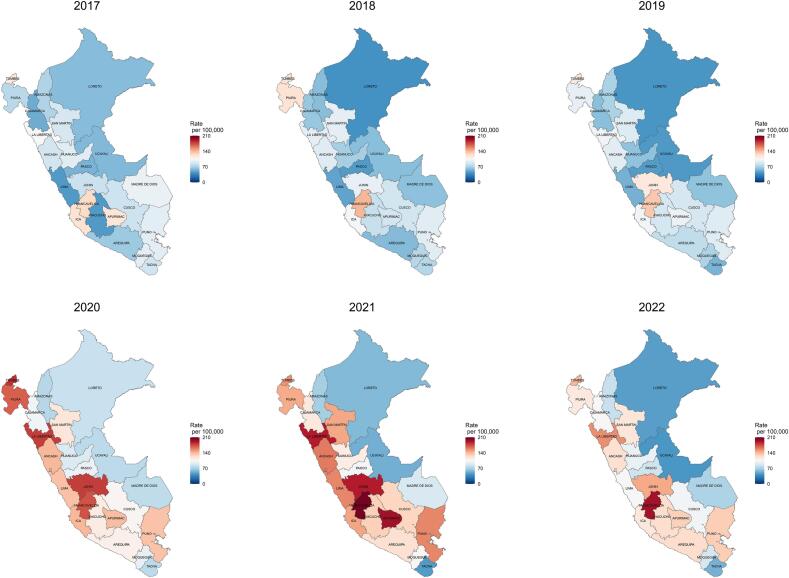
Geographic and temporal profiles of cardiovascular-related mortality in Peru between 2017 and 2022. Colors indicate the age-standardized cardiovascular mortality rates. Lines inside the map indicate the boundaries of the Peruvian regions. Coast; Ica, La Libertad, Lima, Moquegua, Piura, Tacna, Tumbes. Highland; Ancash, Apurimac, Arequipa, Ayacucho, Cajamarca, Cusco, Huancavelica, Huanuco, Junin, Pasco, Puno. Amazon; Amazonas, Loreto, Madre de Dios, San Martin, Ucayali [53].

**Fig. 2:**
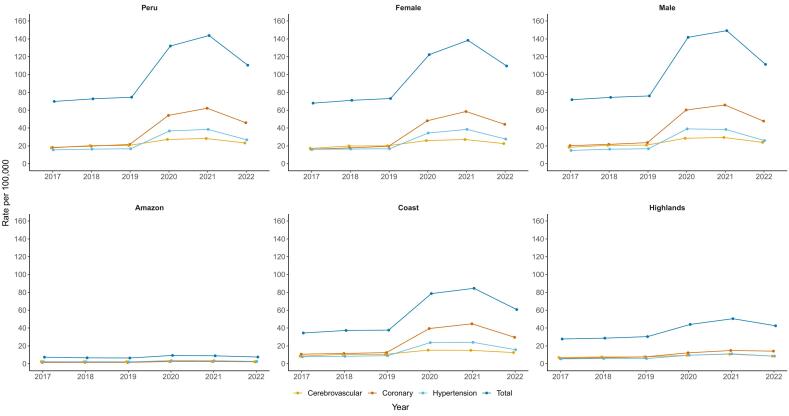
Cardiovascular-related mortality in Peru by natural regions and phenotype between 2017 and 2022. Values are age-standardized cardiovascular mortality rates.

**Fig. 3:**
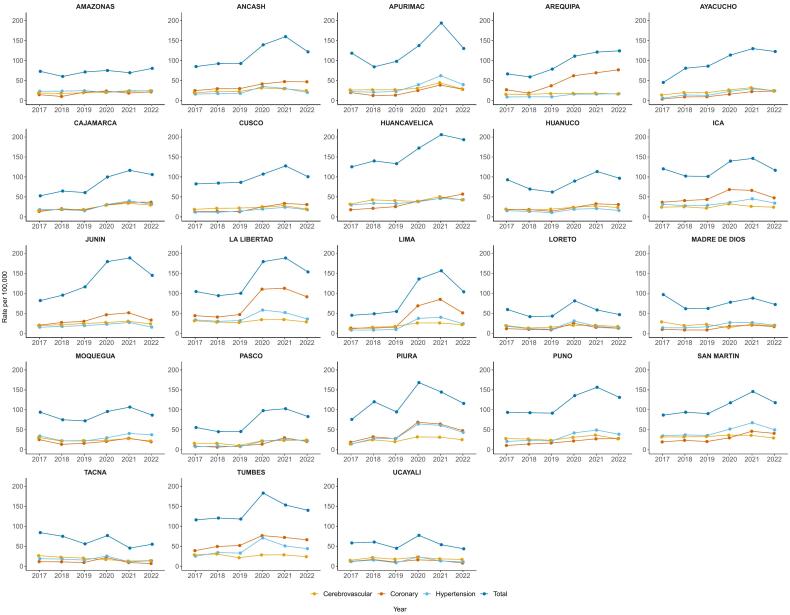
Cardiovascular-related mortality in Peru by regions and phenotype between 2017 and 2022. Values are age-standardized cardiovascular mortality rates.

Between 2017 and 2022, when evaluated by natural regions, the cardiovascular-related mortality rates showed a large increase, mainly during the 2020–2021 period. The increase clustered in the Coast and the central and southern parts of the Highland (Fig. 1). The Coast showed the highest increases in age-standardized cardiovascular-related mortality rates (from 34.5 deaths per 100,000 population in 2017 to 84.6 deaths per 100,000 population in 2021), as compared with the Amazon (from 6.4 deaths per 100,000 population in 2017 to 9.3 deaths per 100,000 population in 2021) and the Highlands (from 27.8 deaths per 100,000 population in 2017 to 50.5 deaths per 100,000 population in 2021) (Fig. 1 and Fig. 2). Similar geographical increments were observed with coronary, hypertensive and cerebrovascular categories (Supplementary Fig. 2, Supplementary Fig. 3, Supplementary Fig. 4, Supplementary Fig. 5). Deaths caused by coronary diseases showed an increase clustered in the Coast, with the highest increases in La Libertad, Lima and Arequipa, while deaths caused by hypertensive diseases showed an increase clustered in the northern Coast and in the east part of the Amazon, with the highest increases in Tumbes, Piura and San Martin. Deaths caused by cerebrovascular diseases showed an increase clustered in the Coast and in the Highlands, with the highest increases in Huancavelica, Apurimac and Puno. When evaluating by regions, our maps showed an increased trend in the age-standardized cardiovascular-related mortality rates in all regions (Fig. 1, Fig. 2, Fig. 3). Between 2017 and 2019 there was a relative steady pattern with the highest rates located mainly in Huancavelica (Highlands), Tumbes (Coast) and Ica (Coast), while between 2020 and 2022 the results showed a large increase in the cardiovascular-related mortality rates (Fig. 1, Fig. 2, Fig. 3). The highest rate increases in this period occurred in 2021 in Huancavelica, Apurimac and Junin, all located in the Highlands (Fig. 1, Fig. 2, Fig. 3).

### 3.3 Time trends

When comparing the periods 2017–2019 and 2020–2022, the age-standardized cardiovascular-related mortality rates increased both at national and regional levels (Supplementary Table 2). Peru showed a change from 72.4 per 100,000 population in 2017–2019 to 128.7 per 100,000 population in 2020–2022, that is a 77.8 % increase. At regional level the major changes occurred in Lima (from 49.6 per 100,000 population in 2017–2019 to 132.1 per 100,000 population in 2020–2022, a 166.2 % increase) followed by Pasco (from 48.9 per 100,000 population in 2017–2019 to 94.6 per 100,000 population in 2020–2022, a 93.5 % increase) and Cajamarca (from 59.5 per 100,000 population in 2017–2019 to 107.5 per 100,000 population in 2020–2022, an 80.6 % increase). The only department that decreased its average age-standardized cardiovascular-related mortality rates between the two periods was Tacna (from 71.6 per 100,000 population in 2017–2019 to 59.0 per 100,000 population in 2020–2022, a 17.6 % decrease).

At national level, age-standardized cardiovascular-related mortality rates ranged from 69.9 deaths per 100,000 population in 2017 to 143.7 deaths per 100,000 population in 2021 (Supplementary Table 3). The rate in 2020 (132.0 deaths per 100,000 population) nearly duplicated that of 2019 (74.6 deaths per 100,000 population) and continued increasing in 2021 (143.7 deaths per 100,000 population) followed by a decrease in 2022 (110.5 deaths per 100,000 population). Similar patterns were observed for cardiovascular-related mortality rates in all regions during the study period. The lowest rate was observed in the department of Loreto (41.8 deaths per 100,000 population) in 2018, while the highest was observed in Huancavelica in 2021 (205.9 deaths per 100,000 population). These regions are located in the Amazon and the Highlands, respectively.

When evaluated by phenotypes, coronary deaths showed the higher increase as compared to hypertensive and cerebrovascular-related deaths. Deaths caused by coronary diseases increased from 18.2 deaths per 100,000 population in 2017 to 62.1 deaths per 100,000 population in 2021, while deaths caused by hypertensive diseases increased from 15.2 deaths per 100,000 population in 2017 to 38.5 deaths per 100,000 population in 2021. Deaths caused by cerebrovascular diseases showed an increase from 17.8 deaths per 100,000 population in 2017 to 28.3 deaths per 100,000 population in 2021. Males as compared to females showed higher age-standardized cardiovascular-related mortality rates during the study period. Male age-standardized cardiovascular-related mortality rates increased from 71.8 deaths per 100,000 population in 2017 to 149.19 deaths per 100,000 population in 2021 while females increased from 67.9 in 2017 to 138.4 in 2021. Age-standardized cardiovascular-related mortality rates in all 23 regions showed an increasing trend since 2020 with different slopes, and a reduction in 2022. The three largest increases were observed in regions located in the Highlands, specifically in Ayacucho (from 45.1 deaths per 100,000 population in 2017 to 122.7 deaths per 100,000 population in 2022), Huancavelica (125.4 deaths per 100,000 population in 2017 to 193.3 deaths per 100,000 population in 2022) and Junin (82.2 deaths per 100,000 population in 2018 to 145.0 deaths per 100,000 population in 2021) (Fig. 1). Males and females showed similar an increasing trend in the study period. However, males showed higher age-standardized cardiovascular-related mortality rates (Supplementary Figs. 2A and 2B).

### 3.4 Equiplots

**Fig. 4:**
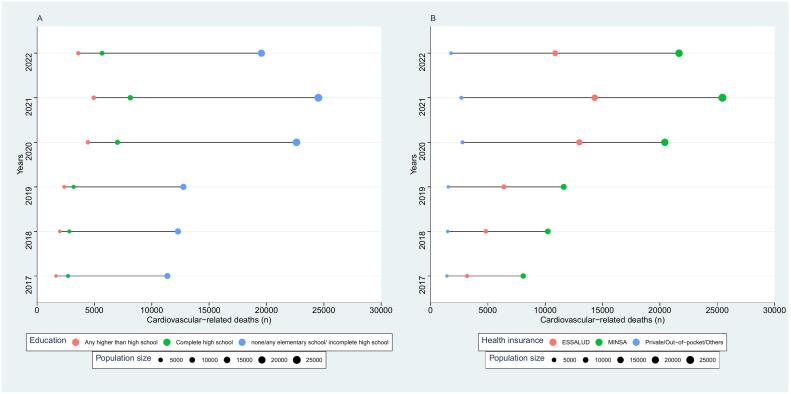
Equiplots of cardiovascular-related mortality according to education attainment (A), health insurance provider (B). The size of the bubbles is relative to the number of births.

Cardiovascular-related mortality was higher in subjects with lower education (Fig. 4A and Table 1). Between 2017 and 2022, cardiovascular-related deaths increased from 11,360 in 2017 to 19,555 in 2022 in subjects with none/any elementary school/incomplete high school education, while in subjects with complete high school education increased from 2692 in 2017 to 5670 deaths in 2022 and in subjects with any higher than high school education cardiovascular-related deaths increased from 1645 in 2017 to 4079 in 2022. The equiplots showed an increased gap between lower and higher educated groups during the study period, with this gap becoming substantially larger since 2020. The gap between subjects with none/any elementary school/incomplete high school and any higher than high school education increased 64 % from 9715 deaths in 2017 to 15,962 deaths in 2022, while the gap between subjects with complete high school and any higher than high school education increased by 98.4 % from 1047 deaths in 2017 to 2077 deaths in 2022. Cardiovascular-related mortality was higher in subjects with public health insurance (Fig. 4B and Table 1). In subjects with SIS insurance, cardiovascular-related deaths increased from 8085 deaths in 2017 to 21,666 deaths in 2022. In subjects with ESSALUD health insurance cardiovascular-related deaths increased from 3193 deaths in 2017 to 10,867 deaths in 2022. In subjects with private/out-of-pocket health expenditure cardiovascular-related deaths increased 1434 in 2017 deaths to 1794 deaths in 2022. The equiplots showed a large gap between private and public health insurance during the study period, with these differences largely increasing since 2020. The gap between subjects with SIS health insurance and private/out-of-pocket health expenditure increased 198.8 % from 6651 deaths to 19,872 deaths in 2022, while the gap between ESSALUD health insurance and private/out-of-pocket health expenditure increased 415.8 from 1759 deaths in 2017 to 9073 deaths in 2022.

### 3.5 Ecological associations

In the linear mixed model analysis, across regions, the Covid-19 pandemic period was associated with higher age-standardized cardiovascular-related mortality rates (mean difference 95%CI 43.78 (29.28, 58.27), as compared with the pre pandemic period), and a higher unemployment rate was associated with higher age-standardized cardiovascular-related mortality rates (5.09 (0.64, 9.53), per percentage increase), while a higher Gini coefficient was associated with lower age-standardized cardiovascular-related mortality rate (−350.33 (−565.59, −135.07) per unit increase) (Table 2).

**Table 2:** Association of socioeconomic variables and age-standardized cardiovascular-related mortality between 2017 and 2021.

Variable	Regression coefficient	95 % CI	*p*-Value
Time	**43.78**	**(29.28, 58.27)**	***p*** **<** **0.001**
GDP per capita	0.00	(−0.01, 0.00)	0.1753
Gini coefficient for income	**−350.33**	**(−565.59, −135.07)**	**0.002**
Urban population (%)	−0.93	(−1.95, 0.09)	0.073
Poverty (%)	0.97	(−0.51, 2.46)	0.196
Average per capita monthly income (USD)	0.23	(−0.08, 0.54)	0.139
Unemployment rate	**5.09**	**(0.64, 9.53)**	**0.026**
Years of schooling (median)	−2.80	(−13.86, 8.26)	0.614
SIS health insurance (%)	−0.57	(−1.84, 0.69)	0.368
Cardiovascular-related outpatient visits rate	0.00	(0.00, 0.00)	0.123

Values represent time-adjusted coefficients and 95 % coefficient interval (CI) between the socioeconomic variables and age-standardized cardiovascular mortality rate. GDP; Gross Domestic Product, SIS; Seguro Integral de Salud, in Spanish. Bold indicates p-value <0.05.

## 4 Discussion

### 4.1 Main results

In 2017–2022, Peru showed an increased trend in cardiovascular-related mortality which markedly increased during the Covid-19 pandemic, and a small decrease in 2022. Males showed a slightly higher age-standardized cardiovascular-related mortality rates than females and both followed a similar increasing trend. The cardiovascular-related mortality clustered in the Coast and Highlands. We identified regions with an alarming increase of cardiovascular-related mortality, highlighting the need for more studies and interventions.

### 4.2 Potential explanations

Age-standardized cardiovascular-related mortality rates show an increased trend overtime, with a more pronounced increase since 2020. Our equiplots showed that the number of deaths concentrated in subjects with lower education and users of public health insurance. It is well established that social determinants of health such as lower education and lower socioeconomic status are predictors of adverse cardiovascular events. The mechanisms of underlying these associations are complex and most probably multifactorial, including poor education, increasing prevalence of unhealthy lifestyle behaviors and lower medication adherence, as well as lower access to health care [30]. Our linear mixed-effects analysis a regional level, showed that, the Covid-19 pandemic period, and higher unemployment were associated with higher cardiovascular-related mortality overtime while a higher Gini coefficient was associated with lower cardiovascular-related mortality overtime. This is in line with previous studies exploring the effect of socioeconomical factors and the Covid-19 pandemic on mortality [13,[31], [32], [33], [34], [35]]. Numerous studies support the role of socioeconomic status in shaping cardiovascular morbidity and mortality [[36], [37], [38]]. Specifically, there is evidence suggesting that income level is independently associated with cardiovascular disease [[39], [40], [41]]. In a study in Boston, among 2097 patients, long-term mortality after myocardial infarction was higher in individuals living in more socioeconomically disadvantaged neighborhoods [31]. Higher prevalence of cardiovascular diseases and deaths in lower income strata may be attributed to psychosocial stressors and coping behaviors such as drug or alcohol abuse [42].

Our analysis of changes in cardiovascular-related mortality between 2017 and 2019 and 2020–2022 showed that all except one department exhibited a substantial excess of cardiovascular-related deaths in the 2020–2022 period. The impact of the Covid-19 pandemic is worth considering, as there is evidence suggesting that the pandemic has disrupted health systems and limited healthcare access for patients with chronic diseases, including those related to the cardiovascular system. This is also supported by previous studies conducted in Peru, which have shown a significant decrease in the number of outpatient consultations during the pandemic [13]. Furthermore, it is important to note that COVID-19 can lead to complications in the cardiovascular system, and therefore the high number of infections in the country may partially account for the increased number of cardiovascular-related deaths observed during the period 2020 to 2022.

Our study identifies areas (e.g. regions in the Coast and Highlands) where interventions are urgently need to reduce cardiovascular-related deaths. The regional differences in cardiovascular-related mortality rates can be influenced by various factors. In terms of socioeconomic disparities, regions within Peru exhibit varying levels of poverty, income inequality, and unemployment rates. Higher levels of poverty and lower socioeconomic status can contribute to increased cardiovascular mortality rates due to limited access to healthcare, unhealthy lifestyle behaviors, and higher stress levels. A cohort previous in Peru found that individuals with lower socioeconomic status had higher cardiovascular mortality rates, highlighting the role of poverty and income inequality in cardiovascular health outcomes [43]. In addition, disparities in healthcare access and infrastructure can impact cardiovascular mortality rates. Urban areas such as Lima have better healthcare facilities, specialized medical services and higher doctor-to-patient ratios, when compared to rural areas. Limited access to timely and appropriate healthcare services can contribute to higher mortality rates in regions with poorer healthcare infrastructure [44]. Furthermore, it is essential to consider the potential impact of the COVID-19 pandemic on cardiovascular mortality rates. The pandemic may have affected different regions of Peru to varying degrees, depending on factors like population density, healthcare capacity, and public health responses. Policies to reduce health inequities focus on regions with higher cardiovascular-related mortality are needed. Moreover, active participation and leadership of different stakeholders including national agencies, local governments, policy makers, healthcare providers and health professional organizations in these endeavor are necessary [45,46]. Disruptions in healthcare services, delayed or inadequate treatment, and increased psychological stress due to the pandemic could also have contributed to higher cardiovascular mortality rates in some areas [47]. Implementation and development of digital health interventions such as telemedicine to enable the continuity of disease monitoring and consultations should be considered, particularly in situations where conventional face-to-face healthcare access is restricted or limited [48,49].

### 4.3 Public health implications

Our study shows an increased trend of cardiovascular-related mortality in Peru, clustered in the Coast and Highlands of Peru, and aggravated by the Covid-19 pandemic. Our results provide information for future surveillance and monitoring of cardiovascular health in Peru. National and local authorities can use our results to design interventions and update current guidelines and policies. Our study suggests that current interventions may have not been sufficient. The gaps between natural regions and regions might imply that locally adapted policies and interventions are need. Moreover, more studies on the potential drivers of age-standardized cardiovascular-related mortality are needed to better approach potential prevention and control interventions.

### 4.4 Strengths and limitations

Our study used a national death registry with information from 2017 to 2022. This large database allowed us the exploration of national and subnational trends and the analysis of some socioeconomical variables. Nevertheless, some limitations need to be addressed. Variations in registration practices across regions may have introduced information bias and cannot be completely ruled out. However, the data registration in the national death registration system adheres to standard procedures and is carried out by trained healthcare professionals following national guidelines [50]. Although the dataset aims to cover the entire territory, the coverage of cause-of-deaths in SINADEF register was previously estimated in 70 % for 2017–2019 [53]. It is possible that some deaths in remote areas were missed. We suspect that missed deaths from the national registry might underestimate our findings and might have influenced certain areas, making then appear less pronounced. This might be the case in areas located in the Amazon region where mortality rates were lower than in other regions. However, the national death registry system has been improving overtime and complements the online registration with manual entries for deaths occurring in areas where internet connection is not available [14]. Although we excluded Lambayeque region due to inconsistences in the number of deaths, our national estimates align closely with previous reports on cardiovascular mortality [51]. The use of a linear mixed model to explore associations between variables at subnational level might not necessarily represent what is happening at individual level (ecological fallacy), but it is a useful tool to monitor health indicators at national and subnational levels and to generate new hypotheses for future studies [52]. Thus, our ecological association should not be interpreted as implying causal associations and more studies at individual level are need to corroborate our findings.

## 5 Conclusion

Overtime, there has been a notable rise in cardiovascular-related mortality in Peru, particularly during the Covid-19 pandemic with a slight decrease in 2022. Gaining a comprehensive understanding of the factors that contribute to the increase in cardiovascular deaths in Peru will facilitate the development of precise interventions at both the national and regional levels.

The following are the supplementary data related to this article.

Supplementary material Image 1

Supplementary Fig. 2 Cardiovascular mortality in Peru by departments and phenotype and sex between 2017 and 2022. Values are age-standardized cardiovascular mortality rates. A) Females B) Males. Supplementary Fig. 2

Supplementary Fig. 3 Geographic and temporal profiles of deaths caused by coronary diseases in Peru between 2017 and 2022.
Colors indicate the age-standardized cardiovascular mortality rates. Lines inside the map indicate the boundaries of the Peruvian regions. Image 2

Supplementary Fig. 4 Geographic and temporal profiles of deaths caused by hypertensive diseases in Peru between 2017 and 2022.
Colors indicate the age-standardized cardiovascular mortality rates. Lines inside the map indicate the boundaries of the Peruvian regions. Image 3

Supplementary Fig. 5 Geographic and temporal profiles of deaths caused by cerebrovascular diseases in Peru between 2017 and 2022.
Colors indicate the age-standardized cardiovascular mortality rates. Lines inside the map indicate the boundaries of the Peruvian regions. Image 4

## Role of funding source

All the authors have full access to the data in the study. The authors have the final responsibility for the decision to submit for publication and for the accuracy of the data. The authors are responsible of the opinions in the manuscript which is do not necessarily represent those of their organizations. The funders of this research had no role in the study design and data collection, data analysis, data interpretation or writing the manuscript.

## Contributors

Conceptualization: HGQP, NSA, KNCT; formal analysis: HGQP, NSA, KNCT, JO; writing original draft: HGQP, NSA, KNCT; writing review: HGQP, NSA, KNCT, JO, TM, LH, CG. All authors approved the submitted version.

## Data sharing statement

Data used in the current study are open access and can be downloaded from the Ministry of Health in Peru: https://www.datosabiertos.gob.pe/dataset/sinadef-certificado-defunciones?_gl=1*1dd9hqh*_ga*ODU3NjM2NDEzLjE2OTc3NDIyNTU.*_ga_NY8L5SJPMB*MTY5Nzc3NDUyMC40LjEuMTY5Nzc3ODYxNC4wLjAuMA, and from the National institute of Statistics and Computing: http://iinei.inei.gob.pe/microdatos/.

## Declaration of competing interest

The authors declare no competing interests. TM is the co-founder of Epistudia, an online learning and evidence synthesis platform. No funding was provided for Epistudia for this project, and Epistudia had no involvement in the design, writing, or interpretation of the results.
