# Supplementary material for: A comprehensive analysis of cardiovascular mortality trends in Peru from 2017 to 2022: Insights from 183,386 deaths of the national death registry
Source: Am Heart J Plus. 2023 Oct 20;35:100335. doi: 10.1016/j.ahjo.2023.100335 (PMC10946053; doi:10.1016/j.ahjo.2023.100335)
Supplement: Supplementary file 1 — Supplementary material [file mmc1.docx]

**Supplementary material**

**A comprehensive analysis of cardiovascular mortality trends in Peru from 2017 to 2022: insights from 183,386 deaths of the national death registry**

**Short title:** cardiovascular-related mortality in Peru

Hugo G Quezada-Pinedo^1,2,3 *^ Noushin Sadat Ahanchi^4,8,9 *^, Kim N Cajachagua-Torres^1,3,5^, Jordan A Obeso-Manrique^3^, Luis Huicho^3,7^, Christoph Gräni^4^ Taulant Muka^6^

*Shared first authorship

^1^The Generation R Study Group, Erasmus MC, University Medical Center Rotterdam, Rotterdam, The Netherlands

^2^Department of Cardiology, Bern University Hospital, University of Bern, Bern, Switzerland.

^3^Centro de Investigación en Salud Materna e Infantil and Centro de Investigación para el Desarrollo Integral y Sostenible, Universidad Peruana Cayetano Heredia, Lima, Peru

^4^Institute of Social and Preventive Medicine (ISPM), Graduate School of Health Sciences, University of Bern, Bern, Switzerland.

^5^Harvard T.H. Chan School of Public Health, Boston, MA, USA.

^6^Epistudia, Bern, Switzerland.

^7^Facultad de Medicina “Alberto Hurtado”, Universidad Peruana Cayetano Heredia, Lima, Peru

^8^Graduate School for Health Sciences, University of Bern, Bern, Switzerland

^9^Department of Internal Medicine, Internal Medicine, Lausanne University Hospital, Lausanne, Switzerland

**Contents**

[Supplementary Table 1. 3](#_Toc148653216)

[Supplementary Table 2. 4](#_Toc148653217)

[Supplementary Table 3. 5](#_Toc148653218)

[Supplementary Figure 1. 15](#_Toc148653219)

[Supplementary Figure 2. 16](#_Toc148653220)

[STROBE checklist. 17](#_Toc148653221)

[Abstract in Spanish 19](#_Toc148653222)

**Supplementary Table 1. Poverty percentage across regions in Peru 2017-2021.**

| **Regions** | **2015** | **2016** | **2017** | **2018** | **2019** | **2020** | **2021** |
| --- | --- | --- | --- | --- | --- | --- | --- |
| AMAZONAS | 42.3 | 39.7 | 34.9 | 33.5 | 30.5 | 36.1 | 30.1 |
| ANCASH | 24.0 | 21.7 | 22.4 | 20.3 | 17.5 | 29.8 | 21.9 |
| APURIMAC | 38.7 | 38.2 | 35.9 | 31.8 | 29.1 | 35.5 | 28.3 |
| AREQUIPA | 8.2 | 8.3 | 8.1 | 8.6 | 6.0 | 18.6 | 12.0 |
| AYACUCHO | 40.7 | 37.5 | 35.6 | 37.5 | 39.4 | 46.4 | 36.0 |
| CAJAMARCA | 50.9 | 48.2 | 47.5 | 41.9 | 38.0 | 42.5 | 39.7 |
| CUSCO | 17.6 | 20.4 | 25.3 | 22.9 | 23.0 | 32.1 | 22.0 |
| HUANCAVELICA | 45.2 | 44.7 | 38.9 | 38.7 | 36.9 | 47.7 | 41.2 |
| HUANUCO | 35.6 | 32.7 | 34.3 | 29.9 | 29.4 | 42.6 | 35.5 |
| ICA | 5.0 | 3.0 | 3.3 | 3.1 | 2.6 | 8.5 | 6.6 |
| JUNIN | 19.1 | 17.5 | 21.2 | 21.5 | 20.7 | 31.4 | 26.4 |
| LA LIBERTAD | 25.9 | 24.5 | 23.5 | 20.8 | 24.7 | 31.9 | 26.8 |
| LAMBAYEQUE | 20.8 | 14.8 | 18.5 | 12.0 | 10.2 | 15.8 | 14.0 |
| LIMA | 11.5 | 11.1 | 13.2 | 12.9 | 14.2 | 26.6 | 24.6 |
| LORETO | 35.0 | 34.2 | 35.3 | 32.7 | 32.2 | 33.1 | 34.6 |
| MADRE DE DIOS | 7.2 | 7.5 | 4.6 | 3.2 | 9.2 | 11.3 | 7.7 |
| MOQUEGUA | 7.8 | 9.6 | 9.2 | 8.7 | 9.2 | 18.2 | 10.2 |
| PASCO | 38.5 | 35.8 | 38.6 | 35.0 | 30.3 | 44.8 | 42.1 |
| PIURA | 29.4 | 30.7 | 28.7 | 27.5 | 24.2 | 35.0 | 25.3 |
| PUNO | 34.6 | 34.8 | 32.7 | 37.0 | 34.7 | 42.5 | 42.6 |
| SAN MARTIN | 27.6 | 23.5 | 26.1 | 24.9 | 25.4 | 26.0 | 20.3 |
| TACNA | 15.1 | 14.6 | 13.9 | 13.6 | 13.3 | 23.3 | 21.8 |
| TUMBES | 13.0 | 11.9 | 11.8 | 10.9 | 13.7 | 32.6 | 19.3 |
| UCAYALI | 11.5 | 12.7 | 13.9 | 10.6 | 12.3 | 21.2 | 17.0 |

Source: INEI (Instituto Nacional de Estadística e Informatica, in Spanish)

**Supplementary Table 2. Age-standardized cardiovascular mortality rates change in Peruvian departments between 2017 and 2022**

| **Department** | **2017** | **2018** | **2019** | **2020** | **2021** | **2022** | **Average**  **2017-2019** | **Average**  **2020-2022** | **Change** | **Change %** |
| --- | --- | --- | --- | --- | --- | --- | --- | --- | --- | --- |
| Peru | 69.9 | 72.7 | 74.6 | 132.0 | 143.7 | 110.5 | 72.4 | 128.7 | 56.3 | 77.8 |
| Amazonas | 72.9 | 60.2 | 71.5 | 75.2 | 69.5 | 80.5 | 68.2 | 75.1 | 6.8 | 10.0 |
| Ancash | 85.0 | 92.3 | 92.7 | 139.4 | 159.9 | 121.8 | 90.0 | 140.4 | 50.4 | 56.0 |
| Apurimac | 118.4 | 84.3 | 98.0 | 137.5 | 193.9 | 130.2 | 100.2 | 153.9 | 53.6 | 53.5 |
| Arequipa | 66.6 | 59.1 | 78.6 | 111.0 | 121.3 | 124.3 | 68.1 | 118.9 | 50.8 | 74.6 |
| Ayacucho | 45.1 | 80.9 | 86.1 | 113.8 | 129.9 | 122.7 | 70.7 | 122.1 | 51.4 | 72.7 |
| Cajamarca | 52.9 | 64.9 | 60.9 | 99.9 | 116.5 | 106.1 | 59.5 | 107.5 | 48.0 | 80.6 |
| Cusco | 82.8 | 84.8 | 86.5 | 107.3 | 127.7 | 100.7 | 84.7 | 111.9 | 27.2 | 32.1 |
| Huancavelica | 125.4 | 140.2 | 133.2 | 172.4 | 205.9 | 193.3 | 132.9 | 190.5 | 57.6 | 43.3 |
| Huanuco | 93.0 | 69.8 | 62.3 | 89.8 | 113.6 | 96.8 | 75.0 | 100.1 | 25.0 | 33.4 |
| Ica | 120.3 | 102.2 | 101.3 | 139.7 | 146.5 | 116.7 | 108.0 | 134.3 | 26.4 | 24.4 |
| Junin | 82.0 | 95.6 | 116.4 | 179.6 | 188.7 | 145.0 | 98.0 | 171.1 | 73.1 | 74.6 |
| La Libertad | 104.6 | 94.3 | 100.3 | 179.5 | 188.6 | 153.7 | 99.7 | 173.9 | 74.2 | 74.4 |
| Lima | 45.2 | 49.0 | 54.8 | 136.0 | 156.6 | 103.9 | 49.6 | 132.1 | 82.5 | 166.2 |
| Loreto | 59.6 | 41.8 | 43.3 | 81.3 | 58.5 | 46.9 | 48.2 | 62.3 | 14.0 | 29.1 |
| Madre de Dios | 97.0 | 61.7 | 62.4 | 77.9 | 88.1 | 72.2 | 73.7 | 79.4 | 5.7 | 7.8 |
| Moquegua | 94.0 | 75.1 | 72.3 | 95.9 | 106.8 | 86.6 | 80.5 | 96.4 | 16.0 | 19.8 |
| Pasco | 55.7 | 45.3 | 45.7 | 98.0 | 102.8 | 83.1 | 48.9 | 94.6 | 45.7 | 93.5 |
| Piura | 75.9 | 120.1 | 94.8 | 168.1 | 144.4 | 115.9 | 96.9 | 142.8 | 45.9 | 47.3 |
| Puno | 93.5 | 92.7 | 91.7 | 135.4 | 156.4 | 131.1 | 92.6 | 141.0 | 48.4 | 52.2 |
| San martin | 86.8 | 94.0 | 90.5 | 117.7 | 145.8 | 117.9 | 90.4 | 127.1 | 36.7 | 40.6 |
| Tacna | 84.0 | 74.8 | 55.9 | 76.7 | 45.3 | 54.9 | 71.6 | 59.0 | -12.6 | -17.6 |
| Tumbes | 115.9 | 120.5 | 118.0 | 182.9 | 153.0 | 139.8 | 118.1 | 158.6 | 40.4 | 34.2 |
| Ucayali | 58.1 | 60.4 | 44.7 | 77.2 | 53.8 | 43.5 | 54.4 | 58.1 | 3.7 | 6.8 |

**Supplementary Table 3. Age-standardized cardiovascular mortality rates and 95%CI by phenotype and sex in Peru between 2017 and 2022**

| Region | Sex | Phenotype | 2017 | 2018 | 2019 | 2020 | 2021 | 2022 |
| --- | --- | --- | --- | --- | --- | --- | --- | --- |
| **Country** |  |  |  |  |  |  |  |  |
| PERU | Female | Cerebrovascular | 17 (16, 18) | 20 (19, 21) | 20 (19, 21) | 26 (25, 27) | 27 (26, 28) | 23 (22, 23) |
| PERU | Female | Coronary | 16 (16, 17) | 18 (17, 19) | 20 (19, 20) | 48 (47, 49) | 59 (57, 60) | 44 (43, 45) |
| PERU | Female | Hypertension | 16 (15, 17) | 16 (16, 17) | 17 (16, 17) | 34 (34, 35) | 38 (38, 39) | 28 (27, 28) |
| PERU | Female | Total | 68 (67, 69) | 71 (70, 73) | 73 (72, 74) | 122 (121, 124) | 138 (137, 140) | 110 (108, 111) |
| PERU | Male | Cerebrovascular | 18 (18, 19) | 21 (20, 21) | 21 (20, 22) | 29 (28, 29) | 29 (29, 30) | 24 (23, 25) |
| PERU | Male | Coronary | 20 (20, 21) | 22 (21, 22) | 24 (23, 24) | 60 (59, 61) | 66 (65, 67) | 48 (47, 49) |
| PERU | Male | Hypertension | 15 (14, 16) | 16 (16, 17) | 17 (16, 17) | 39 (38, 40) | 38 (37, 39) | 26 (25, 27) |
| PERU | Male | Total | 72 (70, 73) | 74 (73, 76) | 76 (75, 77) | 142 (140, 144) | 149 (147, 151) | 111 (110, 113) |
| PERU | Total | Cerebrovascular | 18 (17, 18) | 20 (20, 21) | 20 (20, 21) | 27 (27, 28) | 28 (28, 29) | 23 (23, 24) |
| PERU | Total | Coronary | 18 (18, 19) | 20 (19, 20) | 22 (21, 22) | 54 (53, 55) | 62 (61, 63) | 46 (45, 47) |
| PERU | Total | Hypertension | 16 (15, 16) | 16 (16, 17) | 17 (16, 17) | 37 (36, 37) | 38 (38, 39) | 27 (26, 27) |
| PERU | Total | Total | 70 (69, 71) | 73 (72, 74) | 75 (74, 76) | 132 (131, 133) | 144 (142, 145) | 110 (109, 112) |
| **Natural region** |  |  |  |  |  |  |  |  |
| AMAZON | Total | Total | 7 (7, 8) | 7 (6, 7) | 6 (6, 7) | 9 (9, 10) | 9 (9, 9) | 7 (7, 8) |
| COAST | Total | Total | 34 (34, 35) | 37 (37, 38) | 38 (37, 38) | 79 (78, 80) | 85 (84, 86) | 61 (60, 62) |
| HIGHLANDS | Total | Total | 28 (27, 28) | 29 (28, 29) | 30 (30, 31) | 44 (43, 45) | 50 (50, 51) | 43 (42, 43) |
| **Department** |  |  |  |  |  |  |  |  |
| AMAZONAS | Female | Cerebrovascular | 20 (13, 26) | 20 (14, 27) | 21 (14, 27) | 19 (13, 24) | 27 (20, 33) | 18 (12, 24) |
| AMAZONAS | Female | Coronary | 12 (7, 17) | 10 (6, 15) | 22 (15, 28) | 19 (13, 25) | 19 (14, 25) | 20 (14, 26) |
| AMAZONAS | Female | Hypertension | 25 (18, 32) | 26 (19, 33) | 28 (20, 35) | 16 (11, 22) | 31 (24, 38) | 21 (15, 27) |
| AMAZONAS | Female | Total | 69 (57, 81) | 63 (52, 74) | 77 (65, 89) | 64 (53, 75) | 76 (65, 88) | 75 (64, 87) |
| AMAZONAS | Male | Cerebrovascular | 18 (12, 24) | 15 (10, 21) | 18 (12, 24) | 22 (16, 28) | 20 (14, 25) | 31 (24, 38) |
| AMAZONAS | Male | Coronary | 17 (12, 23) | 10 (6, 14) | 20 (14, 26) | 28 (21, 35) | 18 (12, 23) | 23 (17, 29) |
| AMAZONAS | Male | Hypertension | 22 (15, 28) | 21 (15, 27) | 22 (16, 28) | 26 (19, 32) | 19 (13, 24) | 26 (20, 33) |
| AMAZONAS | Male | Total | 76 (64, 89) | 58 (48, 68) | 67 (56, 78) | 86 (74, 98) | 63 (53, 73) | 86 (74, 97) |
| AMAZONAS | Total | Cerebrovascular | 19 (14, 23) | 18 (14, 22) | 19 (15, 24) | 20 (16, 25) | 23 (19, 27) | 25 (20, 29) |
| AMAZONAS | Total | Coronary | 15 (11, 18) | 10 (7, 13) | 21 (16, 25) | 24 (19, 28) | 19 (15, 23) | 22 (17, 26) |
| AMAZONAS | Total | Hypertension | 23 (18, 28) | 24 (19, 28) | 25 (20, 29) | 21 (17, 25) | 25 (20, 29) | 24 (19, 28) |
| AMAZONAS | Total | Total | 73 (64, 81) | 60 (53, 68) | 72 (63, 80) | 75 (67, 83) | 70 (62, 77) | 80 (72, 89) |
| ANCASH | Female | Cerebrovascular | 19 (15, 23) | 19 (16, 23) | 22 (18, 26) | 28 (24, 32) | 29 (25, 34) | 24 (20, 28) |
| ANCASH | Female | Coronary | 26 (22, 30) | 24 (20, 29) | 28 (23, 32) | 38 (33, 43) | 44 (39, 49) | 46 (41, 52) |
| ANCASH | Female | Hypertension | 18 (15, 22) | 18 (14, 22) | 20 (16, 23) | 33 (29, 38) | 32 (27, 36) | 22 (18, 25) |
| ANCASH | Female | Total | 87 (79, 95) | 88 (80, 96) | 92 (84, 100) | 128 (119, 137) | 154 (144, 164) | 122 (113, 130) |
| ANCASH | Male | Cerebrovascular | 19 (15, 23) | 27 (23, 32) | 23 (19, 27) | 35 (30, 39) | 30 (25, 34) | 26 (22, 30) |
| ANCASH | Male | Coronary | 24 (19, 28) | 35 (30, 40) | 32 (27, 36) | 45 (40, 50) | 51 (45, 56) | 47 (42, 53) |
| ANCASH | Male | Hypertension | 14 (11, 17) | 17 (13, 20) | 16 (12, 19) | 37 (32, 42) | 29 (25, 33) | 18 (15, 22) |
| ANCASH | Male | Total | 83 (75, 91) | 97 (89, 105) | 93 (85, 101) | 151 (141, 161) | 166 (156, 176) | 122 (113, 131) |
| ANCASH | Total | Cerebrovascular | 19 (16, 22) | 23 (20, 26) | 23 (20, 25) | 31 (28, 35) | 30 (26, 33) | 25 (22, 28) |
| ANCASH | Total | Coronary | 25 (22, 28) | 30 (26, 33) | 30 (27, 33) | 41 (38, 45) | 47 (43, 51) | 47 (43, 51) |
| ANCASH | Total | Hypertension | 16 (14, 19) | 17 (15, 20) | 18 (15, 20) | 35 (32, 39) | 30 (27, 33) | 20 (18, 23) |
| ANCASH | Total | Total | 85 (79, 91) | 92 (87, 98) | 93 (87, 98) | 139 (133, 146) | 160 (153, 167) | 122 (116, 128) |
| APURIMAC | Female | Cerebrovascular | 28 (20, 35) | 30 (22, 37) | 31 (24, 39) | 37 (28, 45) | 48 (39, 57) | 26 (19, 32) |
| APURIMAC | Female | Coronary | 20 (14, 26) | 12 (7, 16) | 12 (7, 17) | 23 (16, 29) | 44 (35, 52) | 23 (16, 29) |
| APURIMAC | Female | Hypertension | 26 (19, 34) | 19 (13, 26) | 26 (19, 32) | 46 (37, 56) | 73 (62, 84) | 40 (32, 48) |
| APURIMAC | Female | Total | 123 (107, 138) | 84 (71, 97) | 103 (89, 117) | 148 (132, 164) | 211 (192, 230) | 128 (113, 142) |
| APURIMAC | Male | Cerebrovascular | 25 (18, 32) | 24 (17, 31) | 23 (17, 29) | 25 (18, 31) | 41 (33, 50) | 32 (25, 40) |
| APURIMAC | Male | Coronary | 20 (14, 26) | 13 (8, 18) | 15 (10, 20) | 27 (20, 34) | 34 (26, 42) | 33 (26, 41) |
| APURIMAC | Male | Hypertension | 18 (12, 24) | 22 (16, 28) | 20 (14, 26) | 33 (25, 40) | 52 (42, 61) | 39 (31, 48) |
| APURIMAC | Male | Total | 114 (100, 129) | 84 (72, 97) | 93 (80, 106) | 127 (113, 142) | 178 (160, 195) | 133 (118, 147) |
| APURIMAC | Total | Cerebrovascular | 26 (21, 31) | 27 (22, 32) | 27 (22, 32) | 30 (25, 36) | 44 (38, 51) | 29 (24, 34) |
| APURIMAC | Total | Coronary | 20 (15, 24) | 12 (9, 16) | 13 (10, 17) | 25 (20, 30) | 39 (33, 44) | 28 (23, 33) |
| APURIMAC | Total | Hypertension | 22 (18, 27) | 21 (16, 25) | 23 (18, 27) | 40 (34, 45) | 62 (55, 69) | 40 (34, 45) |
| APURIMAC | Total | Total | 118 (108, 129) | 84 (75, 93) | 98 (89, 107) | 138 (127, 148) | 194 (181, 207) | 130 (120, 141) |
| AREQUIPA | Female | Cerebrovascular | 15 (12, 18) | 16 (13, 19) | 18 (15, 21) | 16 (14, 19) | 20 (17, 23) | 18 (15, 21) |
| AREQUIPA | Female | Coronary | 24 (20, 28) | 17 (14, 20) | 34 (30, 39) | 57 (52, 63) | 67 (62, 73) | 77 (71, 83) |
| AREQUIPA | Female | Hypertension | 10 (8, 13) | 9 (7, 11) | 11 (8, 13) | 16 (14, 19) | 15 (13, 18) | 19 (16, 22) |
| AREQUIPA | Female | Total | 67 (61, 73) | 59 (53, 64) | 80 (73, 86) | 105 (98, 112) | 118 (111, 126) | 129 (121, 137) |
| AREQUIPA | Male | Cerebrovascular | 16 (13, 19) | 16 (13, 19) | 16 (13, 19) | 19 (16, 22) | 17 (14, 20) | 14 (12, 17) |
| AREQUIPA | Male | Coronary | 30 (25, 34) | 21 (17, 24) | 39 (35, 44) | 67 (61, 73) | 72 (66, 78) | 76 (70, 82) |
| AREQUIPA | Male | Hypertension | 7 (5, 9) | 9 (7, 12) | 8 (6, 10) | 16 (13, 19) | 17 (14, 20) | 15 (12, 17) |
| AREQUIPA | Male | Total | 66 (60, 72) | 59 (54, 65) | 78 (71, 84) | 117 (110, 125) | 124 (116, 132) | 119 (112, 127) |
| AREQUIPA | Total | Cerebrovascular | 16 (13, 18) | 16 (14, 18) | 17 (15, 19) | 18 (16, 20) | 19 (16, 21) | 16 (14, 18) |
| AREQUIPA | Total | Coronary | 27 (24, 30) | 19 (16, 21) | 37 (34, 40) | 62 (58, 66) | 69 (65, 73) | 77 (73, 81) |
| AREQUIPA | Total | Hypertension | 9 (7, 11) | 9 (8, 11) | 9 (8, 11) | 16 (14, 18) | 16 (14, 18) | 17 (15, 19) |
| AREQUIPA | Total | Total | 67 (62, 71) | 59 (55, 63) | 79 (74, 83) | 111 (106, 116) | 121 (116, 127) | 124 (119, 130) |
| AYACUCHO | Female | Cerebrovascular | 13 (9, 18) | 22 (17, 27) | 22 (17, 27) | 28 (22, 34) | 38 (31, 44) | 28 (23, 34) |
| AYACUCHO | Female | Coronary | 3 (1, 5) | 10 (7, 14) | 10 (6, 13) | 16 (12, 20) | 23 (18, 29) | 25 (20, 31) |
| AYACUCHO | Female | Hypertension | 7 (4, 10) | 16 (12, 21) | 16 (12, 20) | 26 (20, 31) | 35 (29, 42) | 27 (22, 33) |
| AYACUCHO | Female | Total | 48 (40, 55) | 96 (85, 107) | 100 (89, 111) | 119 (107, 131) | 146 (133, 159) | 134 (122, 147) |
| AYACUCHO | Male | Cerebrovascular | 14 (10, 18) | 18 (13, 22) | 17 (12, 21) | 25 (20, 30) | 27 (21, 32) | 21 (17, 26) |
| AYACUCHO | Male | Coronary | 4 (2, 6) | 8 (5, 11) | 9 (6, 12) | 15 (11, 20) | 21 (16, 26) | 22 (17, 27) |
| AYACUCHO | Male | Hypertension | 5 (3, 8) | 12 (8, 16) | 10 (6, 13) | 20 (16, 25) | 24 (19, 29) | 22 (17, 26) |
| AYACUCHO | Male | Total | 43 (36, 50) | 67 (58, 75) | 73 (64, 82) | 109 (98, 120) | 114 (103, 126) | 112 (101, 123) |
| AYACUCHO | Total | Cerebrovascular | 14 (11, 17) | 20 (16, 23) | 19 (16, 23) | 26 (23, 30) | 32 (28, 36) | 25 (21, 28) |
| AYACUCHO | Total | Coronary | 4 (2, 5) | 9 (7, 11) | 9 (7, 12) | 16 (13, 19) | 22 (19, 26) | 24 (20, 27) |
| AYACUCHO | Total | Hypertension | 6 (4, 8) | 14 (11, 17) | 13 (10, 16) | 23 (19, 27) | 29 (25, 33) | 24 (21, 28) |
| AYACUCHO | Total | Total | 45 (40, 50) | 81 (74, 88) | 86 (79, 93) | 114 (106, 122) | 130 (121, 138) | 123 (114, 131) |
| CAJAMARCA | Female | Cerebrovascular | 16 (13, 19) | 19 (16, 22) | 20 (17, 23) | 29 (25, 33) | 37 (32, 41) | 28 (24, 32) |
| CAJAMARCA | Female | Coronary | 13 (10, 16) | 20 (16, 23) | 18 (15, 21) | 31 (27, 35) | 35 (31, 39) | 35 (31, 39) |
| CAJAMARCA | Female | Hypertension | 22 (18, 25) | 19 (16, 22) | 16 (13, 19) | 31 (27, 35) | 45 (40, 50) | 34 (30, 38) |
| CAJAMARCA | Female | Total | 53 (48, 59) | 64 (58, 70) | 64 (58, 70) | 102 (94, 109) | 118 (110, 126) | 107 (100, 114) |
| CAJAMARCA | Male | Cerebrovascular | 15 (12, 18) | 19 (16, 22) | 17 (14, 20) | 30 (26, 34) | 32 (28, 36) | 32 (28, 36) |
| CAJAMARCA | Male | Coronary | 14 (11, 17) | 21 (18, 25) | 17 (14, 20) | 29 (25, 33) | 39 (34, 43) | 39 (34, 43) |
| CAJAMARCA | Male | Hypertension | 15 (12, 18) | 18 (15, 21) | 15 (12, 18) | 31 (27, 35) | 36 (31, 40) | 30 (26, 34) |
| CAJAMARCA | Male | Total | 52 (47, 58) | 66 (60, 72) | 58 (52, 64) | 98 (91, 105) | 115 (107, 123) | 105 (98, 112) |
| CAJAMARCA | Total | Cerebrovascular | 16 (13, 18) | 19 (17, 21) | 19 (16, 21) | 29 (26, 32) | 34 (31, 37) | 30 (27, 32) |
| CAJAMARCA | Total | Coronary | 14 (12, 16) | 20 (18, 23) | 17 (15, 19) | 30 (27, 33) | 37 (34, 40) | 37 (34, 40) |
| CAJAMARCA | Total | Hypertension | 19 (16, 21) | 18 (16, 21) | 15 (13, 17) | 31 (28, 34) | 40 (37, 43) | 32 (29, 35) |
| CAJAMARCA | Total | Total | 53 (49, 57) | 65 (61, 69) | 61 (57, 65) | 100 (95, 105) | 117 (111, 122) | 106 (101, 111) |
| CUSCO | Female | Cerebrovascular | 18 (14, 21) | 22 (18, 26) | 17 (14, 20) | 28 (24, 32) | 28 (24, 32) | 21 (17, 24) |
| CUSCO | Female | Coronary | 12 (9, 14) | 15 (12, 18) | 12 (9, 14) | 25 (21, 29) | 32 (28, 36) | 31 (27, 35) |
| CUSCO | Female | Hypertension | 14 (11, 17) | 13 (11, 16) | 17 (14, 20) | 20 (16, 23) | 23 (19, 26) | 20 (17, 24) |
| CUSCO | Female | Total | 83 (75, 90) | 90 (82, 97) | 82 (75, 89) | 108 (100, 116) | 125 (117, 134) | 104 (97, 112) |
| CUSCO | Male | Cerebrovascular | 20 (17, 24) | 21 (18, 25) | 27 (23, 31) | 20 (17, 24) | 28 (25, 32) | 19 (16, 22) |
| CUSCO | Male | Coronary | 15 (12, 18) | 13 (11, 16) | 14 (11, 17) | 25 (21, 29) | 35 (31, 40) | 31 (27, 35) |
| CUSCO | Male | Hypertension | 10 (7, 12) | 10 (8, 13) | 12 (10, 15) | 19 (16, 23) | 25 (22, 29) | 16 (13, 19) |
| CUSCO | Male | Total | 83 (76, 90) | 80 (73, 87) | 91 (84, 98) | 107 (99, 114) | 130 (121, 138) | 97 (90, 104) |
| CUSCO | Total | Cerebrovascular | 19 (17, 21) | 22 (19, 24) | 22 (20, 25) | 24 (22, 27) | 28 (26, 31) | 20 (18, 22) |
| CUSCO | Total | Coronary | 13 (11, 15) | 14 (12, 16) | 13 (11, 15) | 25 (22, 28) | 34 (31, 37) | 31 (28, 34) |
| CUSCO | Total | Hypertension | 12 (10, 14) | 12 (10, 14) | 15 (13, 17) | 20 (17, 22) | 24 (22, 27) | 18 (16, 20) |
| CUSCO | Total | Total | 83 (78, 88) | 85 (80, 90) | 86 (81, 91) | 107 (102, 113) | 128 (122, 134) | 101 (96, 106) |
| HUANCAVELICA | Female | Cerebrovascular | 35 (26, 44) | 52 (42, 62) | 39 (30, 48) | 38 (29, 47) | 53 (43, 64) | 44 (34, 54) |
| HUANCAVELICA | Female | Coronary | 18 (12, 24) | 25 (18, 33) | 27 (19, 34) | 45 (35, 54) | 49 (39, 59) | 72 (60, 84) |
| HUANCAVELICA | Female | Hypertension | 36 (27, 45) | 47 (37, 57) | 38 (29, 47) | 44 (34, 53) | 45 (36, 55) | 50 (40, 60) |
| HUANCAVELICA | Female | Total | 145 (127, 162) | 166 (147, 184) | 138 (121, 155) | 189 (169, 209) | 208 (188, 229) | 211 (189, 232) |
| HUANCAVELICA | Male | Cerebrovascular | 29 (21, 36) | 34 (25, 42) | 42 (33, 51) | 40 (31, 49) | 48 (38, 58) | 41 (32, 51) |
| HUANCAVELICA | Male | Coronary | 18 (12, 24) | 18 (12, 24) | 25 (18, 32) | 33 (25, 42) | 44 (35, 54) | 43 (33, 52) |
| HUANCAVELICA | Male | Hypertension | 24 (17, 31) | 21 (15, 28) | 29 (21, 36) | 33 (25, 41) | 48 (38, 58) | 36 (27, 45) |
| HUANCAVELICA | Male | Total | 106 (91, 121) | 115 (99, 130) | 128 (112, 145) | 156 (139, 174) | 203 (183, 224) | 176 (157, 196) |
| HUANCAVELICA | Total | Cerebrovascular | 32 (26, 38) | 43 (36, 49) | 41 (34, 47) | 39 (33, 45) | 51 (43, 58) | 43 (36, 49) |
| HUANCAVELICA | Total | Coronary | 18 (14, 22) | 22 (17, 26) | 26 (21, 31) | 39 (33, 45) | 46 (39, 53) | 57 (49, 65) |
| HUANCAVELICA | Total | Hypertension | 30 (24, 36) | 34 (28, 40) | 33 (27, 39) | 38 (32, 44) | 47 (40, 54) | 43 (36, 50) |
| HUANCAVELICA | Total | Total | 125 (114, 137) | 140 (128, 152) | 133 (121, 145) | 172 (159, 186) | 206 (191, 221) | 193 (179, 208) |
| HUANUCO | Female | Cerebrovascular | 20 (16, 25) | 18 (14, 22) | 19 (15, 23) | 23 (18, 28) | 26 (21, 31) | 22 (18, 27) |
| HUANUCO | Female | Coronary | 18 (14, 22) | 20 (15, 25) | 15 (11, 19) | 23 (18, 28) | 30 (25, 36) | 31 (26, 37) |
| HUANUCO | Female | Hypertension | 17 (12, 21) | 15 (11, 19) | 10 (7, 13) | 18 (14, 22) | 21 (16, 25) | 18 (14, 22) |
| HUANUCO | Female | Total | 99 (89, 110) | 73 (64, 81) | 63 (55, 71) | 89 (80, 99) | 106 (95, 116) | 101 (91, 110) |
| HUANUCO | Male | Cerebrovascular | 19 (14, 23) | 16 (12, 20) | 20 (15, 24) | 24 (20, 29) | 30 (24, 35) | 25 (20, 30) |
| HUANUCO | Male | Coronary | 18 (14, 23) | 17 (13, 22) | 14 (10, 17) | 24 (19, 28) | 35 (29, 41) | 31 (25, 36) |
| HUANUCO | Male | Hypertension | 15 (11, 19) | 12 (9, 16) | 12 (8, 15) | 21 (16, 25) | 21 (16, 25) | 14 (11, 18) |
| HUANUCO | Male | Total | 87 (77, 97) | 67 (59, 75) | 62 (54, 69) | 90 (81, 100) | 121 (111, 132) | 93 (84, 103) |
| HUANUCO | Total | Cerebrovascular | 20 (16, 23) | 17 (14, 20) | 20 (16, 23) | 24 (20, 27) | 28 (24, 31) | 24 (20, 27) |
| HUANUCO | Total | Coronary | 18 (15, 21) | 19 (15, 22) | 14 (12, 17) | 24 (20, 27) | 33 (29, 37) | 31 (27, 35) |
| HUANUCO | Total | Hypertension | 16 (13, 19) | 14 (11, 16) | 11 (9, 13) | 19 (16, 22) | 21 (18, 24) | 16 (13, 19) |
| HUANUCO | Total | Total | 93 (86, 100) | 70 (64, 76) | 62 (57, 68) | 90 (83, 96) | 114 (106, 121) | 97 (90, 104) |
| ICA | Female | Cerebrovascular | 23 (19, 28) | 24 (19, 28) | 24 (19, 28) | 30 (25, 35) | 26 (21, 30) | 23 (19, 27) |
| ICA | Female | Coronary | 32 (27, 37) | 38 (32, 44) | 36 (31, 42) | 62 (55, 69) | 61 (54, 67) | 50 (44, 56) |
| ICA | Female | Hypertension | 32 (26, 37) | 27 (22, 32) | 28 (23, 33) | 35 (29, 40) | 44 (38, 50) | 37 (32, 43) |
| ICA | Female | Total | 118 (108, 128) | 99 (90, 108) | 94 (85, 103) | 126 (116, 136) | 141 (130, 151) | 117 (108, 127) |
| ICA | Male | Cerebrovascular | 25 (21, 30) | 27 (23, 32) | 20 (16, 24) | 35 (30, 41) | 27 (23, 32) | 26 (21, 30) |
| ICA | Male | Coronary | 42 (35, 48) | 44 (38, 50) | 51 (45, 58) | 75 (68, 83) | 72 (65, 80) | 46 (40, 51) |
| ICA | Male | Hypertension | 32 (26, 37) | 29 (24, 34) | 30 (25, 35) | 38 (32, 43) | 47 (41, 53) | 32 (27, 36) |
| ICA | Male | Total | 123 (112, 133) | 105 (96, 115) | 109 (99, 118) | 153 (142, 164) | 152 (142, 163) | 116 (107, 125) |
| ICA | Total | Cerebrovascular | 24 (21, 28) | 26 (22, 29) | 22 (19, 25) | 33 (29, 36) | 26 (23, 30) | 24 (21, 27) |
| ICA | Total | Coronary | 37 (33, 41) | 41 (37, 45) | 44 (39, 48) | 69 (63, 74) | 66 (61, 71) | 48 (44, 52) |
| ICA | Total | Hypertension | 32 (28, 36) | 28 (25, 32) | 29 (26, 33) | 36 (32, 40) | 45 (41, 50) | 35 (31, 38) |
| ICA | Total | Total | 120 (113, 128) | 102 (96, 109) | 101 (95, 108) | 140 (132, 147) | 146 (139, 154) | 117 (110, 123) |
| JUNIN | Female | Cerebrovascular | 18 (15, 22) | 23 (19, 26) | 25 (22, 29) | 29 (25, 33) | 32 (28, 36) | 24 (20, 27) |
| JUNIN | Female | Coronary | 18 (15, 21) | 26 (23, 30) | 31 (27, 35) | 44 (39, 49) | 51 (46, 56) | 32 (28, 37) |
| JUNIN | Female | Hypertension | 17 (13, 20) | 19 (16, 22) | 21 (18, 25) | 23 (20, 27) | 28 (24, 32) | 16 (13, 19) |
| JUNIN | Female | Total | 81 (74, 88) | 99 (91, 107) | 123 (114, 131) | 181 (171, 191) | 188 (178, 199) | 151 (142, 160) |
| JUNIN | Male | Cerebrovascular | 21 (17, 24) | 20 (17, 24) | 24 (20, 28) | 25 (22, 29) | 28 (24, 32) | 23 (20, 27) |
| JUNIN | Male | Coronary | 23 (19, 26) | 28 (24, 32) | 29 (25, 33) | 49 (44, 55) | 52 (46, 57) | 34 (30, 38) |
| JUNIN | Male | Hypertension | 14 (11, 17) | 16 (13, 19) | 18 (15, 21) | 22 (19, 26) | 27 (23, 30) | 15 (12, 18) |
| JUNIN | Male | Total | 83 (76, 90) | 92 (85, 99) | 110 (102, 118) | 178 (168, 188) | 189 (179, 199) | 139 (130, 147) |
| JUNIN | Total | Cerebrovascular | 20 (17, 22) | 22 (19, 24) | 25 (22, 27) | 27 (24, 30) | 30 (27, 33) | 24 (21, 26) |
| JUNIN | Total | Coronary | 20 (18, 23) | 27 (24, 30) | 30 (27, 33) | 47 (43, 50) | 52 (48, 55) | 33 (30, 36) |
| JUNIN | Total | Hypertension | 15 (13, 18) | 17 (15, 20) | 20 (17, 22) | 23 (20, 25) | 27 (25, 30) | 16 (14, 18) |
| JUNIN | Total | Total | 82 (77, 87) | 96 (90, 101) | 116 (111, 122) | 180 (172, 187) | 189 (181, 196) | 145 (139, 151) |
| LA LIBERTAD | Female | Cerebrovascular | 32 (29, 36) | 31 (27, 34) | 27 (24, 31) | 30 (27, 34) | 30 (27, 34) | 29 (26, 32) |
| LA LIBERTAD | Female | Coronary | 41 (36, 45) | 37 (33, 41) | 41 (37, 45) | 96 (90, 102) | 102 (96, 108) | 85 (80, 90) |
| LA LIBERTAD | Female | Hypertension | 33 (29, 37) | 30 (27, 34) | 30 (26, 33) | 53 (48, 57) | 50 (46, 54) | 35 (32, 39) |
| LA LIBERTAD | Female | Total | 104 (98, 111) | 95 (89, 102) | 95 (89, 101) | 160 (152, 168) | 176 (168, 184) | 148 (141, 155) |
| LA LIBERTAD | Male | Cerebrovascular | 31 (27, 34) | 25 (22, 29) | 27 (24, 30) | 38 (35, 42) | 39 (35, 42) | 28 (25, 31) |
| LA LIBERTAD | Male | Coronary | 48 (43, 52) | 45 (40, 49) | 53 (48, 57) | 125 (118, 132) | 123 (117, 130) | 98 (92, 104) |
| LA LIBERTAD | Male | Hypertension | 34 (30, 38) | 30 (27, 34) | 34 (30, 38) | 64 (59, 69) | 54 (50, 59) | 37 (33, 40) |
| LA LIBERTAD | Male | Total | 105 (98, 112) | 93 (87, 100) | 106 (99, 112) | 199 (191, 208) | 201 (193, 210) | 159 (152, 167) |
| LA LIBERTAD | Total | Cerebrovascular | 32 (29, 34) | 28 (26, 30) | 27 (25, 29) | 34 (32, 37) | 34 (32, 37) | 29 (26, 31) |
| LA LIBERTAD | Total | Coronary | 44 (41, 47) | 41 (38, 44) | 47 (44, 50) | 110 (106, 115) | 113 (108, 117) | 92 (88, 96) |
| LA LIBERTAD | Total | Hypertension | 34 (31, 36) | 30 (28, 33) | 32 (29, 34) | 58 (55, 62) | 52 (49, 55) | 36 (33, 38) |
| LA LIBERTAD | Total | Total | 105 (100, 109) | 94 (90, 99) | 100 (96, 105) | 180 (174, 185) | 189 (183, 194) | 154 (149, 159) |
| LIMA | Female | Cerebrovascular | 10 (9, 11) | 15 (14, 16) | 17 (16, 18) | 24 (23, 26) | 24 (22, 25) | 20 (19, 22) |
| LIMA | Female | Coronary | 11 (10, 12) | 11 (10, 12) | 14 (13, 15) | 60 (58, 62) | 79 (77, 81) | 49 (47, 50) |
| LIMA | Female | Hypertension | 8 (7, 9) | 8 (7, 9) | 10 (9, 10) | 34 (33, 36) | 39 (37, 40) | 25 (23, 26) |
| LIMA | Female | Total | 42 (40, 44) | 45 (43, 47) | 53 (51, 55) | 122 (119, 125) | 147 (144, 150) | 101 (98, 103) |
| LIMA | Male | Cerebrovascular | 12 (11, 13) | 16 (15, 17) | 17 (16, 19) | 28 (26, 29) | 29 (27, 30) | 23 (22, 24) |
| LIMA | Male | Coronary | 16 (14, 17) | 15 (14, 17) | 17 (16, 18) | 78 (76, 81) | 91 (89, 94) | 54 (52, 56) |
| LIMA | Male | Hypertension | 8 (8, 9) | 9 (8, 9) | 10 (9, 11) | 41 (39, 43) | 42 (40, 43) | 24 (22, 25) |
| LIMA | Male | Total | 49 (47, 51) | 53 (51, 55) | 56 (54, 59) | 151 (148, 154) | 167 (163, 170) | 107 (104, 110) |
| LIMA | Total | Cerebrovascular | 11 (10, 12) | 15 (15, 16) | 17 (16, 18) | 26 (25, 27) | 26 (25, 27) | 22 (21, 22) |
| LIMA | Total | Coronary | 13 (12, 14) | 13 (12, 14) | 15 (15, 16) | 69 (67, 70) | 85 (83, 87) | 51 (50, 53) |
| LIMA | Total | Hypertension | 8 (8, 9) | 8 (8, 9) | 10 (9, 10) | 37 (36, 39) | 40 (39, 41) | 24 (23, 25) |
| LIMA | Total | Total | 45 (44, 47) | 49 (48, 50) | 55 (53, 56) | 136 (134, 138) | 157 (154, 159) | 104 (102, 106) |
| LORETO | Female | Cerebrovascular | 18 (14, 22) | 15 (11, 18) | 16 (13, 20) | 17 (13, 20) | 18 (14, 21) | 16 (12, 19) |
| LORETO | Female | Coronary | 7 (5, 10) | 7 (5, 10) | 7 (4, 9) | 21 (17, 25) | 11 (8, 14) | 10 (7, 12) |
| LORETO | Female | Hypertension | 17 (13, 21) | 13 (10, 17) | 11 (8, 14) | 23 (19, 28) | 18 (14, 22) | 10 (8, 13) |
| LORETO | Female | Total | 51 (44, 58) | 40 (35, 46) | 40 (35, 46) | 69 (62, 77) | 51 (45, 57) | 40 (34, 45) |
| LORETO | Male | Cerebrovascular | 21 (17, 25) | 12 (9, 15) | 15 (11, 18) | 23 (19, 27) | 22 (18, 26) | 19 (15, 22) |
| LORETO | Male | Coronary | 16 (12, 19) | 12 (9, 15) | 11 (8, 13) | 29 (25, 34) | 18 (15, 22) | 16 (13, 19) |
| LORETO | Male | Hypertension | 18 (14, 22) | 10 (7, 13) | 10 (7, 13) | 38 (33, 43) | 17 (14, 20) | 16 (13, 19) |
| LORETO | Male | Total | 68 (60, 75) | 43 (37, 49) | 46 (40, 52) | 93 (84, 101) | 66 (59, 72) | 54 (48, 60) |
| LORETO | Total | Cerebrovascular | 20 (17, 22) | 13 (11, 15) | 15 (13, 18) | 20 (17, 23) | 20 (17, 23) | 17 (15, 20) |
| LORETO | Total | Coronary | 12 (9, 14) | 10 (8, 12) | 9 (7, 11) | 25 (22, 28) | 15 (13, 17) | 13 (11, 15) |
| LORETO | Total | Hypertension | 18 (15, 20) | 12 (9, 14) | 10 (8, 12) | 31 (28, 34) | 17 (15, 20) | 13 (11, 16) |
| LORETO | Total | Total | 60 (55, 65) | 42 (38, 46) | 43 (39, 47) | 81 (76, 87) | 59 (54, 63) | 47 (43, 51) |
| MADRE DE DIOS | Female | Cerebrovascular | 19 (8, 30) | 15 (6, 24) | 14 (5, 23) | 12 (4, 19) | 17 (8, 26) | 16 (8, 24) |
| MADRE DE DIOS | Female | Coronary | 8 (1, 15) | 6 (0, 12) | 3 (0, 7) | 9 (2, 16) | 18 (9, 28) | 10 (4, 17) |
| MADRE DE DIOS | Female | Hypertension | 12 (3, 21) | 9 (2, 16) | 17 (7, 26) | 30 (18, 42) | 24 (13, 35) | 22 (12, 31) |
| MADRE DE DIOS | Female | Total | 77 (55, 99) | 60 (42, 79) | 53 (36, 70) | 70 (51, 89) | 70 (52, 89) | 63 (46, 80) |
| MADRE DE DIOS | Male | Cerebrovascular | 36 (23, 49) | 23 (13, 33) | 29 (18, 40) | 16 (8, 24) | 27 (17, 36) | 22 (13, 30) |
| MADRE DE DIOS | Male | Coronary | 11 (4, 18) | 10 (4, 17) | 13 (6, 20) | 25 (15, 35) | 23 (14, 32) | 22 (13, 31) |
| MADRE DE DIOS | Male | Hypertension | 15 (7, 24) | 16 (8, 25) | 16 (8, 24) | 25 (15, 35) | 29 (19, 39) | 19 (11, 28) |
| MADRE DE DIOS | Male | Total | 112 (89, 135) | 62 (45, 78) | 70 (53, 87) | 84 (66, 102) | 102 (83, 121) | 79 (63, 96) |
| MADRE DE DIOS | Total | Cerebrovascular | 29 (20, 38) | 20 (13, 27) | 23 (15, 30) | 14 (9, 20) | 22 (16, 29) | 19 (13, 25) |
| MADRE DE DIOS | Total | Coronary | 10 (5, 15) | 8 (4, 13) | 8 (4, 13) | 18 (12, 24) | 21 (14, 27) | 17 (11, 22) |
| MADRE DE DIOS | Total | Hypertension | 14 (8, 20) | 14 (8, 20) | 16 (10, 23) | 27 (19, 35) | 27 (19, 34) | 20 (14, 27) |
| MADRE DE DIOS | Total | Total | 97 (81, 113) | 62 (49, 74) | 62 (50, 74) | 78 (65, 91) | 88 (75, 102) | 72 (60, 84) |
| MOQUEGUA | Female | Cerebrovascular | 35 (22, 48) | 25 (14, 36) | 23 (13, 33) | 18 (9, 26) | 30 (19, 41) | 20 (11, 29) |
| MOQUEGUA | Female | Coronary | 26 (15, 38) | 11 (4, 18) | 16 (8, 25) | 21 (11, 30) | 27 (17, 38) | 20 (11, 29) |
| MOQUEGUA | Female | Hypertension | 41 (26, 55) | 16 (7, 24) | 21 (11, 30) | 29 (18, 40) | 44 (30, 57) | 42 (29, 54) |
| MOQUEGUA | Female | Total | 99 (76, 121) | 74 (55, 92) | 77 (58, 95) | 87 (68, 106) | 115 (93, 136) | 90 (71, 108) |
| MOQUEGUA | Male | Cerebrovascular | 27 (16, 38) | 19 (10, 28) | 23 (14, 33) | 29 (19, 39) | 26 (16, 35) | 24 (15, 33) |
| MOQUEGUA | Male | Coronary | 25 (14, 35) | 16 (8, 24) | 16 (8, 24) | 21 (12, 30) | 30 (20, 41) | 20 (12, 28) |
| MOQUEGUA | Male | Hypertension | 29 (18, 40) | 28 (17, 39) | 23 (14, 33) | 30 (20, 41) | 38 (27, 50) | 34 (23, 45) |
| MOQUEGUA | Male | Total | 90 (70, 110) | 76 (59, 94) | 68 (52, 85) | 103 (84, 123) | 100 (81, 118) | 84 (67, 101) |
| MOQUEGUA | Total | Cerebrovascular | 31 (22, 39) | 22 (15, 29) | 23 (16, 30) | 24 (17, 30) | 28 (21, 35) | 22 (16, 28) |
| MOQUEGUA | Total | Coronary | 25 (18, 33) | 14 (8, 19) | 16 (10, 22) | 21 (14, 27) | 29 (21, 36) | 20 (14, 26) |
| MOQUEGUA | Total | Hypertension | 34 (25, 43) | 22 (15, 29) | 22 (15, 29) | 30 (22, 38) | 41 (32, 49) | 38 (29, 46) |
| MOQUEGUA | Total | Total | 94 (79, 109) | 75 (62, 88) | 72 (60, 85) | 96 (82, 110) | 107 (93, 121) | 87 (74, 99) |
| PASCO | Female | Cerebrovascular | 19 (11, 26) | 20 (12, 27) | 13 (7, 19) | 22 (14, 31) | 23 (15, 31) | 34 (24, 43) |
| PASCO | Female | Coronary | 7 (2, 11) | 9 (4, 14) | 10 (5, 15) | 16 (9, 22) | 31 (22, 41) | 23 (15, 31) |
| PASCO | Female | Hypertension | 6 (2, 10) | 11 (5, 17) | 11 (5, 16) | 19 (11, 26) | 28 (19, 36) | 30 (21, 39) |
| PASCO | Female | Total | 54 (41, 67) | 52 (40, 65) | 49 (37, 61) | 92 (76, 108) | 103 (86, 120) | 97 (80, 113) |
| PASCO | Male | Cerebrovascular | 14 (7, 20) | 13 (7, 19) | 9 (4, 13) | 22 (14, 29) | 24 (16, 32) | 16 (9, 22) |
| PASCO | Male | Coronary | 12 (6, 18) | 4 (1, 8) | 9 (4, 15) | 13 (7, 18) | 29 (20, 38) | 19 (12, 26) |
| PASCO | Male | Hypertension | 9 (4, 15) | 8 (3, 12) | 5 (1, 9) | 24 (16, 32) | 24 (16, 32) | 16 (10, 23) |
| PASCO | Male | Total | 57 (44, 70) | 39 (28, 49) | 43 (32, 53) | 104 (87, 120) | 103 (86, 119) | 70 (57, 84) |
| PASCO | Total | Cerebrovascular | 16 (11, 21) | 16 (11, 21) | 11 (7, 15) | 22 (17, 28) | 23 (18, 29) | 24 (19, 30) |
| PASCO | Total | Coronary | 9 (5, 13) | 7 (3, 10) | 10 (6, 13) | 14 (10, 18) | 30 (24, 36) | 21 (15, 26) |
| PASCO | Total | Hypertension | 8 (4, 11) | 9 (6, 13) | 8 (4, 11) | 22 (16, 27) | 26 (20, 32) | 23 (17, 28) |
| PASCO | Total | Total | 56 (46, 65) | 45 (37, 53) | 46 (38, 54) | 98 (86, 110) | 103 (91, 115) | 83 (73, 94) |
| PIURA | Female | Cerebrovascular | 13 (11, 16) | 23 (20, 26) | 17 (15, 20) | 31 (28, 35) | 29 (25, 32) | 24 (22, 27) |
| PIURA | Female | Coronary | 17 (14, 20) | 28 (25, 32) | 25 (22, 28) | 54 (49, 58) | 59 (55, 64) | 42 (39, 46) |
| PIURA | Female | Hypertension | 14 (12, 17) | 24 (21, 27) | 26 (23, 29) | 59 (54, 64) | 59 (54, 63) | 43 (39, 47) |
| PIURA | Female | Total | 72 (66, 77) | 111 (104, 118) | 87 (82, 93) | 145 (138, 152) | 136 (129, 143) | 110 (103, 116) |
| PIURA | Male | Cerebrovascular | 16 (13, 18) | 27 (24, 30) | 23 (20, 26) | 33 (29, 36) | 34 (31, 38) | 26 (23, 29) |
| PIURA | Male | Coronary | 21 (18, 24) | 37 (33, 40) | 31 (28, 34) | 84 (78, 90) | 70 (65, 75) | 53 (49, 58) |
| PIURA | Male | Hypertension | 14 (12, 17) | 31 (28, 35) | 31 (28, 35) | 70 (65, 75) | 63 (58, 68) | 44 (40, 48) |
| PIURA | Male | Total | 80 (74, 86) | 129 (122, 136) | 102 (96, 108) | 191 (182, 199) | 153 (145, 160) | 122 (116, 129) |
| PIURA | Total | Cerebrovascular | 15 (13, 16) | 25 (23, 27) | 20 (18, 22) | 32 (30, 35) | 32 (29, 34) | 25 (23, 27) |
| PIURA | Total | Coronary | 19 (17, 21) | 32 (30, 35) | 28 (26, 30) | 69 (65, 72) | 64 (61, 68) | 48 (45, 51) |
| PIURA | Total | Hypertension | 14 (13, 16) | 28 (25, 30) | 28 (26, 31) | 65 (61, 68) | 61 (58, 64) | 44 (41, 46) |
| PIURA | Total | Total | 76 (72, 80) | 120 (115, 125) | 95 (90, 99) | 168 (163, 174) | 144 (139, 149) | 116 (111, 120) |
| PUNO | Female | Cerebrovascular | 27 (23, 31) | 25 (21, 29) | 23 (19, 27) | 30 (26, 35) | 37 (32, 42) | 27 (23, 31) |
| PUNO | Female | Coronary | 10 (7, 12) | 14 (11, 17) | 17 (14, 20) | 21 (17, 25) | 24 (20, 27) | 27 (23, 31) |
| PUNO | Female | Hypertension | 21 (17, 24) | 25 (21, 29) | 24 (20, 28) | 44 (38, 49) | 52 (46, 57) | 43 (38, 48) |
| PUNO | Female | Total | 91 (83, 99) | 93 (85, 100) | 96 (88, 104) | 134 (125, 143) | 150 (140, 159) | 136 (127, 145) |
| PUNO | Male | Cerebrovascular | 29 (25, 34) | 29 (25, 33) | 24 (20, 28) | 32 (27, 36) | 37 (32, 41) | 27 (23, 31) |
| PUNO | Male | Coronary | 12 (9, 15) | 15 (12, 18) | 17 (14, 21) | 23 (19, 27) | 31 (27, 36) | 29 (25, 33) |
| PUNO | Male | Hypertension | 21 (17, 25) | 22 (18, 25) | 20 (16, 23) | 41 (36, 46) | 47 (42, 52) | 35 (30, 39) |
| PUNO | Male | Total | 96 (88, 104) | 93 (85, 100) | 87 (80, 95) | 137 (128, 147) | 163 (153, 173) | 126 (117, 135) |
| PUNO | Total | Cerebrovascular | 28 (25, 31) | 27 (24, 30) | 24 (21, 26) | 31 (28, 34) | 37 (33, 40) | 27 (24, 30) |
| PUNO | Total | Coronary | 11 (9, 13) | 14 (12, 17) | 17 (15, 19) | 22 (19, 25) | 27 (24, 30) | 28 (25, 31) |
| PUNO | Total | Hypertension | 21 (18, 24) | 23 (21, 26) | 22 (19, 25) | 42 (39, 46) | 49 (46, 53) | 39 (35, 42) |
| PUNO | Total | Total | 94 (88, 99) | 93 (87, 98) | 92 (86, 97) | 135 (129, 142) | 156 (150, 163) | 131 (125, 137) |
| SAN MARTIN | Female | Cerebrovascular | 33 (27, 39) | 29 (23, 34) | 32 (26, 37) | 35 (29, 40) | 34 (28, 39) | 29 (24, 33) |
| SAN MARTIN | Female | Coronary | 16 (12, 20) | 23 (18, 27) | 18 (13, 22) | 26 (21, 31) | 44 (38, 50) | 39 (33, 44) |
| SAN MARTIN | Female | Hypertension | 38 (32, 45) | 38 (32, 44) | 33 (28, 39) | 49 (43, 56) | 73 (65, 81) | 48 (42, 55) |
| SAN MARTIN | Female | Total | 85 (75, 94) | 89 (79, 98) | 85 (76, 94) | 108 (98, 118) | 145 (134, 156) | 114 (104, 123) |
| SAN MARTIN | Male | Cerebrovascular | 31 (26, 36) | 35 (30, 41) | 34 (29, 39) | 38 (33, 44) | 38 (33, 44) | 30 (26, 35) |
| SAN MARTIN | Male | Coronary | 23 (18, 27) | 24 (20, 29) | 23 (19, 28) | 33 (28, 39) | 48 (42, 54) | 43 (38, 49) |
| SAN MARTIN | Male | Hypertension | 32 (26, 37) | 36 (31, 42) | 38 (33, 44) | 54 (47, 61) | 63 (56, 70) | 51 (45, 57) |
| SAN MARTIN | Male | Total | 89 (79, 98) | 99 (90, 108) | 95 (87, 104) | 126 (116, 136) | 147 (136, 157) | 122 (112, 131) |
| SAN MARTIN | Total | Cerebrovascular | 32 (28, 36) | 32 (28, 36) | 33 (29, 37) | 37 (33, 40) | 36 (32, 40) | 30 (26, 33) |
| SAN MARTIN | Total | Coronary | 20 (17, 23) | 24 (20, 27) | 21 (18, 24) | 30 (26, 33) | 46 (42, 51) | 41 (37, 45) |
| SAN MARTIN | Total | Hypertension | 35 (31, 39) | 37 (33, 41) | 36 (32, 40) | 52 (47, 56) | 68 (63, 73) | 50 (45, 54) |
| SAN MARTIN | Total | Total | 87 (80, 93) | 94 (87, 101) | 90 (84, 97) | 118 (111, 125) | 146 (138, 153) | 118 (111, 125) |
| TACNA | Female | Cerebrovascular | 27 (18, 35) | 25 (17, 32) | 21 (14, 28) | 13 (8, 18) | 14 (9, 19) | 12 (7, 16) |
| TACNA | Female | Coronary | 7 (3, 11) | 11 (6, 16) | 6 (3, 10) | 17 (11, 22) | 9 (5, 13) | 4 (1, 7) |
| TACNA | Female | Hypertension | 20 (13, 28) | 21 (14, 28) | 13 (7, 18) | 22 (16, 29) | 13 (8, 18) | 13 (8, 18) |
| TACNA | Female | Total | 83 (68, 97) | 82 (68, 95) | 56 (45, 67) | 66 (54, 78) | 42 (33, 52) | 51 (41, 61) |
| TACNA | Male | Cerebrovascular | 26 (18, 34) | 20 (13, 27) | 19 (13, 26) | 20 (14, 27) | 12 (7, 17) | 16 (11, 21) |
| TACNA | Male | Coronary | 16 (10, 22) | 11 (6, 16) | 12 (7, 17) | 27 (19, 34) | 9 (5, 13) | 9 (5, 14) |
| TACNA | Male | Hypertension | 17 (10, 24) | 14 (9, 20) | 18 (12, 24) | 28 (21, 36) | 8 (4, 12) | 14 (9, 19) |
| TACNA | Male | Total | 85 (71, 100) | 68 (56, 81) | 55 (45, 66) | 87 (74, 100) | 48 (38, 58) | 59 (48, 69) |
| TACNA | Total | Cerebrovascular | 26 (21, 32) | 22 (17, 28) | 20 (16, 25) | 17 (13, 21) | 13 (9, 16) | 14 (10, 18) |
| TACNA | Total | Coronary | 11 (8, 15) | 11 (7, 14) | 9 (6, 12) | 22 (17, 26) | 9 (6, 12) | 7 (4, 9) |
| TACNA | Total | Hypertension | 19 (14, 24) | 18 (13, 22) | 15 (11, 19) | 25 (20, 30) | 11 (8, 14) | 14 (10, 17) |
| TACNA | Total | Total | 84 (74, 94) | 75 (66, 84) | 56 (48, 64) | 77 (68, 85) | 45 (39, 52) | 55 (48, 62) |
| TUMBES | Female | Cerebrovascular | 25 (15, 34) | 24 (15, 33) | 18 (10, 26) | 26 (17, 36) | 27 (18, 36) | 27 (18, 36) |
| TUMBES | Female | Coronary | 42 (29, 55) | 52 (39, 66) | 35 (24, 46) | 61 (47, 75) | 59 (45, 72) | 60 (47, 73) |
| TUMBES | Female | Hypertension | 33 (21, 44) | 37 (25, 48) | 28 (18, 38) | 72 (57, 87) | 52 (39, 65) | 46 (35, 58) |
| TUMBES | Female | Total | 123 (101, 145) | 125 (104, 146) | 97 (79, 115) | 173 (149, 197) | 134 (113, 154) | 140 (120, 161) |
| TUMBES | Male | Cerebrovascular | 32 (21, 42) | 36 (26, 47) | 24 (15, 32) | 30 (21, 39) | 30 (21, 39) | 21 (14, 29) |
| TUMBES | Male | Coronary | 36 (25, 47) | 47 (34, 59) | 66 (52, 80) | 90 (74, 106) | 83 (68, 98) | 72 (58, 85) |
| TUMBES | Male | Hypertension | 19 (11, 27) | 32 (22, 42) | 37 (26, 47) | 69 (56, 83) | 49 (38, 61) | 42 (31, 52) |
| TUMBES | Male | Total | 110 (91, 129) | 117 (98, 136) | 136 (116, 156) | 191 (168, 214) | 170 (148, 191) | 139 (121, 158) |
| TUMBES | Total | Cerebrovascular | 28 (21, 35) | 31 (23, 38) | 21 (15, 27) | 28 (22, 35) | 28 (22, 35) | 24 (18, 30) |
| TUMBES | Total | Coronary | 39 (30, 47) | 49 (40, 58) | 52 (43, 61) | 77 (66, 87) | 72 (62, 82) | 66 (57, 76) |
| TUMBES | Total | Hypertension | 25 (18, 32) | 34 (26, 42) | 33 (26, 40) | 71 (60, 81) | 51 (42, 59) | 44 (36, 52) |
| TUMBES | Total | Total | 116 (101, 130) | 121 (106, 135) | 118 (104, 132) | 183 (166, 199) | 153 (138, 168) | 140 (126, 154) |
| UCAYALI | Female | Cerebrovascular | 10 (6, 14) | 17 (12, 22) | 12 (8, 16) | 19 (14, 24) | 14 (10, 19) | 15 (11, 19) |
| UCAYALI | Female | Coronary | 12 (7, 16) | 11 (7, 15) | 7 (4, 10) | 11 (8, 15) | 13 (9, 17) | 6 (4, 9) |
| UCAYALI | Female | Hypertension | 12 (7, 16) | 14 (9, 18) | 8 (5, 12) | 18 (13, 22) | 12 (8, 16) | 12 (9, 16) |
| UCAYALI | Female | Total | 53 (43, 62) | 45 (37, 54) | 38 (31, 46) | 65 (56, 75) | 46 (39, 54) | 38 (32, 45) |
| UCAYALI | Male | Cerebrovascular | 19 (14, 24) | 26 (20, 32) | 22 (17, 28) | 26 (21, 32) | 22 (17, 27) | 19 (14, 24) |
| UCAYALI | Male | Coronary | 12 (8, 17) | 22 (16, 27) | 14 (10, 18) | 20 (15, 25) | 15 (11, 20) | 9 (6, 12) |
| UCAYALI | Male | Hypertension | 11 (7, 15) | 17 (12, 22) | 9 (6, 13) | 28 (22, 34) | 14 (10, 19) | 9 (6, 12) |
| UCAYALI | Male | Total | 63 (54, 73) | 74 (64, 85) | 51 (42, 59) | 88 (78, 98) | 60 (52, 69) | 48 (41, 55) |
| UCAYALI | Total | Cerebrovascular | 15 (11, 18) | 22 (18, 26) | 17 (14, 21) | 23 (19, 27) | 18 (15, 22) | 17 (14, 20) |
| UCAYALI | Total | Coronary | 12 (9, 15) | 17 (13, 20) | 10 (8, 13) | 16 (13, 19) | 14 (11, 17) | 8 (6, 10) |
| UCAYALI | Total | Hypertension | 12 (8, 15) | 15 (12, 19) | 9 (6, 11) | 23 (19, 27) | 13 (10, 16) | 11 (8, 13) |
| UCAYALI | Total | Total | 58 (51, 65) | 60 (54, 67) | 45 (39, 50) | 77 (70, 84) | 54 (48, 59) | 43 (38, 49) |

**Supplementary Figure 1. Flow chart of the participants included in the analysis**

**Sample = 706,885 deaths in 2017-2022**

Complete data in cause of death

**Sample = 190,806 cardiovascular deaths in 2017-2022**

Complete data in cause of death

**Sample = 183,402 cardiovascular deaths**

Excluding Lambayeque

2017 = 18,530

2018 = 20,370

2019 = 21,934

2020 = 40,549

2021 = 45,756

2022 = 36,263

**Sample = 183,386 cardiovascular deaths**

Complete data in age and geography variables

**Total sample = 183,386 cardiovascular deaths**

Hypertensive deaths : 45,991

Coronary deaths : 68,269

Cerebrovascular deaths : 41,380

**Supplementary Figure 2. Cardiovascular mortality in Peru by departments and** phenotype **and sex** between 2017 and 2022

Values are age-standardized cardiovascular mortality rates and 95%C. A) Females B) Males.

**Supplementary Figure 3. Geographic and temporal profiles of deaths caused by coronary diseases in Peru between 2017 and 2022.**

Colors indicate the age-standardized cardiovascular mortality rates. Lines inside the map indicate the boundaries of the Peruvian regions

**Supplementary Figure 4. Geographic and temporal profiles of deaths caused by hypertensive diseases in Peru between 2017 and 2022.**

Colors indicate the age-standardized cardiovascular mortality rates. Lines inside the map indicate the boundaries of the Peruvian regions

**Supplementary Figure 5. Geographic and temporal profiles of deaths caused by cerebrovascular diseases in Peru between 2017 and 2022.**

Colors indicate the age-standardized cardiovascular mortality rates. Lines inside the map indicate the boundaries of the Peruvian regions

**Strengthening the Reporting of Observational studies in Epidemiology (STROBE) checklist.**

STROBE Statement—Checklist of items that should be included in reports of ***cohort studies***

|  | | Item No | Recommendation | Page No |
| --- | --- | --- | --- | --- |
| **Title and abstract** | | 1 | (*a*) Indicate the study’s design with a commonly used term in the title or the abstract | Pages3 |
|  |  |  | (*b*) Provide in the abstract an informative and balanced summary of what was done and what was found |  |
| Introduction | | | | |
| Background/rationale | | 2 | Explain the scientific background and rationale for the investigation being reported | Page7 |
| Objectives | | 3 | State specific objectives, including any prespecified hypotheses | Page8 |
| Methods | | | | |
| Study design | | 4 | Present key elements of study design early in the paper | Page 8 |
| Setting | | 5 | Describe the setting, locations, and relevant dates, including periods of recruitment, exposure, follow-up, and data collection | Page 9 |
| Participants | | 6 | (*a*) Give the eligibility criteria, and the sources and methods of selection of participants. Describe methods of follow-up |  |
|  |  |  | (*b*) For matched studies, give matching criteria and number of exposed and unexposed | Page 9 |
| Variables | | 7 | Clearly define all outcomes, exposures, predictors, potential confounders, and effect modifiers. Give diagnostic criteria, if applicable | Page 8-9 |
| Data sources/ measurement | | 8* | For each variable of interest, give sources of data and details of methods of assessment (measurement). Describe comparability of assessment methods if there is more than one group | Page 8 |
| Bias | | 9 | Describe any efforts to address potential sources of bias | Page 8 |
| Study size | | 10 | Explain how the study size was arrived at | Page 12 |
| Quantitative variables | | 11 | Explain how quantitative variables were handled in the analyses. If applicable, describe which groupings were chosen and why | Page 8-9 |
| Statistical methods | | 12 | (*a*) Describe all statistical methods, including those used to control for confounding | Page 10-11 |
|  |  |  | (*b*) Describe any methods used to examine subgroups and interactions |  |
|  |  |  | (*c*) Explain how missing data were addressed |  |
|  |  |  | (*d*) If applicable, explain how loss to follow-up was addressed |  |
|  |  |  | (*e*) Describe any sensitivity analyses |  |
| Results | | | |  |
| Participants | | 13* | (a) Report numbers of individuals at each stage of study—eg numbers potentially eligible, examined for eligibility, confirmed eligible, included in the study, completing follow-up, and analysed | Page 11-12 |
|  |  |  | (b) Give reasons for non-participation at each stage |  |
|  |  |  | (c) Consider use of a flow diagram |  |
| Descriptive data | | 14* | (a) Give characteristics of study participants (eg demographic, clinical, social) and information on exposures and potential confounders | Page 12  Table 1 |
|  |  |  | (b) Indicate number of participants with missing data for each variable of interest |  |
|  |  |  | (c) Summarise follow-up time (eg, average and total amount) |  |
| Outcome data | | 15* | Report numbers of outcome events or summary measures over time | Table 1 |
| Main results | 16 | (*a*) Give unadjusted estimates and, if applicable, confounder-adjusted estimates and their precision (eg, 95% confidence interval). Make clear which confounders were adjusted for and why they were included | | Table 2 and figure 1,2,3,4 |
|  |  | (*b*) Report category boundaries when continuous variables were categorized | |  |
|  |  | (*c*) If relevant, consider translating estimates of relative risk into absolute risk for a meaningful time period | |  |
| Other analyses | 17 | Report other analyses done—eg analyses of subgroups and interactions, and sensitivity analyses | | Supplementary file |
| Discussion | | | | |
| Key results | 18 | Summarise key results with reference to study objectives | | Page 16 |
| Limitations | 19 | Discuss limitations of the study, taking into account sources of potential bias or imprecision. Discuss both direction and magnitude of any potential bias | | Page 19 |
| Interpretation | 20 | Give a cautious overall interpretation of results considering objectives, limitations, multiplicity of analyses, results from similar studies, and other relevant evidence | | Page 16-18 |
| Generalizability | 21 | Discuss the generalizability (external validity) of the study results | | Page 19 |
| Other information | | | | |
| Funding | 22 | Give the source of funding and the role of the funders for the present study and, if applicable, for the original study on which the present article is based | | Page 21 |

*Give information separately for exposed and unexposed groups.

**Note:** An Explanation and Elaboration article discusses each checklist item and gives methodological background and published examples of transparent reporting. The STROBE checklist is best used in conjunction with this article (freely available on the Web sites of PLoS Medicine at http://www.plosmedicine.org/, Annals of Internal Medicine at http://www.annals.org/, and Epidemiology at http://www.epidem.com/). Information on the STROBE Initiative is available at http://www.strobe-statement.org.

**Abstract in Spanish**

**Antecedentes/Objetivos** Las enfermedades cardiovasculares son la principal causa de mortalidad a nivel mundial y un importante factor de discapacidad. En Perú, existe una falta de estudios sistemáticos sobre la mortalidad relacionada con enfermedades cardiovasculares a nivel nacional y subnacional. Nuestro objetivo fue explorar las tendencias en la mortalidad relacionada a enfermedades cardiovasculares entre 2017 y 2022 en Perú, tanto a nivel nacional como subnacional.

**Sujetos/Métodos** Se utilizaron datos del registro de defunciones peruano 2017-2022. Usando los códigos CIE-10, la mortalidad se clasifico como relacionada a enfermedad hipertensiva, coronaria y cerebrovascular. Se estimaron las tasas de mortalidad estandarizadas por edad de causas cardiovasculares por sexo a nivel nacional, departamental, y por regiones naturales (Costa, Sierra, Amazonía). Estimamos el cambio en las tasas de mortalidad entre 2017-2019 y 2020-2022 y exploramos los factores que contribuyeron al cambio. Exploramos relaciones ecológicas entre las tasas de mortalidad e indicadores socioeconómicos.

**Hallazgos** Se identifico 183,386 muertes relacionadas con enfermedades cardiovasculares. Las muertes relacionadas con enfermedades coronarias (37.2%), fueron seguidas de las muertes relacionadas a enfermedades hipertensivas (25.1%) y de las relacionadas a enfermedades cerebrovasculares (22.6%). Perú mostró una tendencia marcadamente creciente en la mortalidad relacionada con enfermedades cardiovasculares en 2020-2022 (77.8%). El incremento se concentró en la Costa y Sierra con el mayor cambio observado en Lima (132,1%). La más alta mortalidad se encontró en sujetos con menor educación y en sujetos con seguro de salud pública. El coeficiente Gini se asoció con menores tasas de mortalidad mientras que el desempleo se asoció con mayores tasas de mortalidad.

**Interpretación** Se observo un aumento notable en la mortalidad relacionada con enfermedades cardiovasculares en Perú, particularmente durante la pandemia de Covid-19, aunque hubo una leve disminución en 2022. Obtener una comprensión integral de los factores que contribuyen al aumento de las muertes cardiovasculares en Perú facilitará el desarrollo de intervenciones precisas tanto a nivel nacional como departamental.
