# Supplementary material for: A comprehensive analysis of cardiovascular mortality trends in Peru from 2017 to 2022: Insights from 183,386 deaths of the national death registry
Source: Am Heart J Plus. 2023 Oct 20;35:100335. doi: 10.1016/j.ahjo.2023.100335 (PMC10946053; doi:10.1016/j.ahjo.2023.100335)

Supplementary Figure 2 A. Cardiovascular mortality in Peru by departments and phenotype and sex between 2017 and 2022

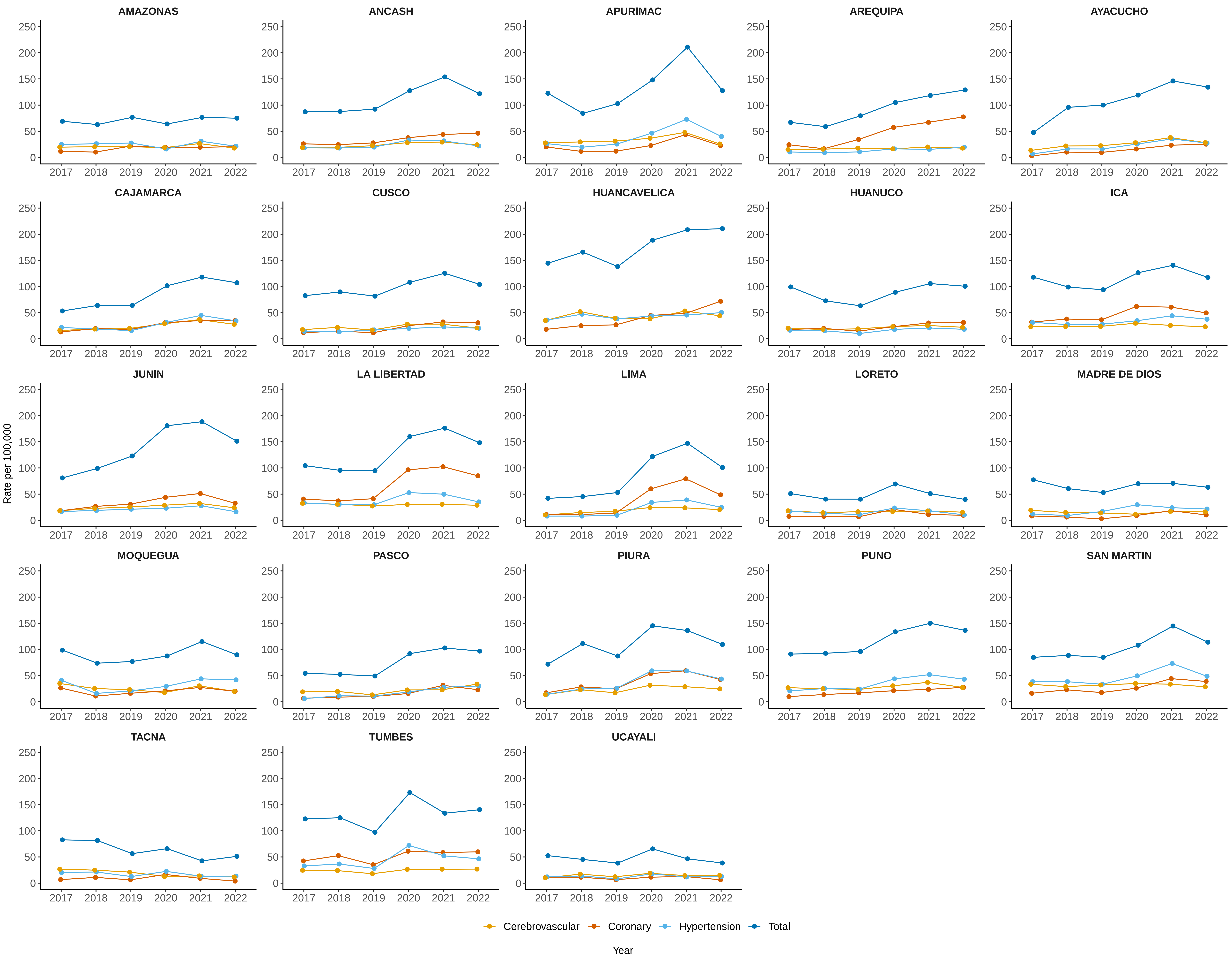

Supplement: Supplementary Fig. 2 — Cardiovascular mortality in Peru by departments and phenotype and sex between 2017 and 2022. Values are age-standardized cardiovascular mortality rates. A) Females B) Males. [file mmc2.pdf]
