# Supplementary material for: A comprehensive analysis of cardiovascular mortality trends in Peru from 2017 to 2022: Insights from 183,386 deaths of the national death registry
Source: Am Heart J Plus. 2023 Oct 20;35:100335. doi: 10.1016/j.ahjo.2023.100335 (PMC10946053; doi:10.1016/j.ahjo.2023.100335)

Supplementary Figure 5. Geographic and temporal profiles of deaths caused by cerebrovascular diseases in Peru between 2017 and 2022

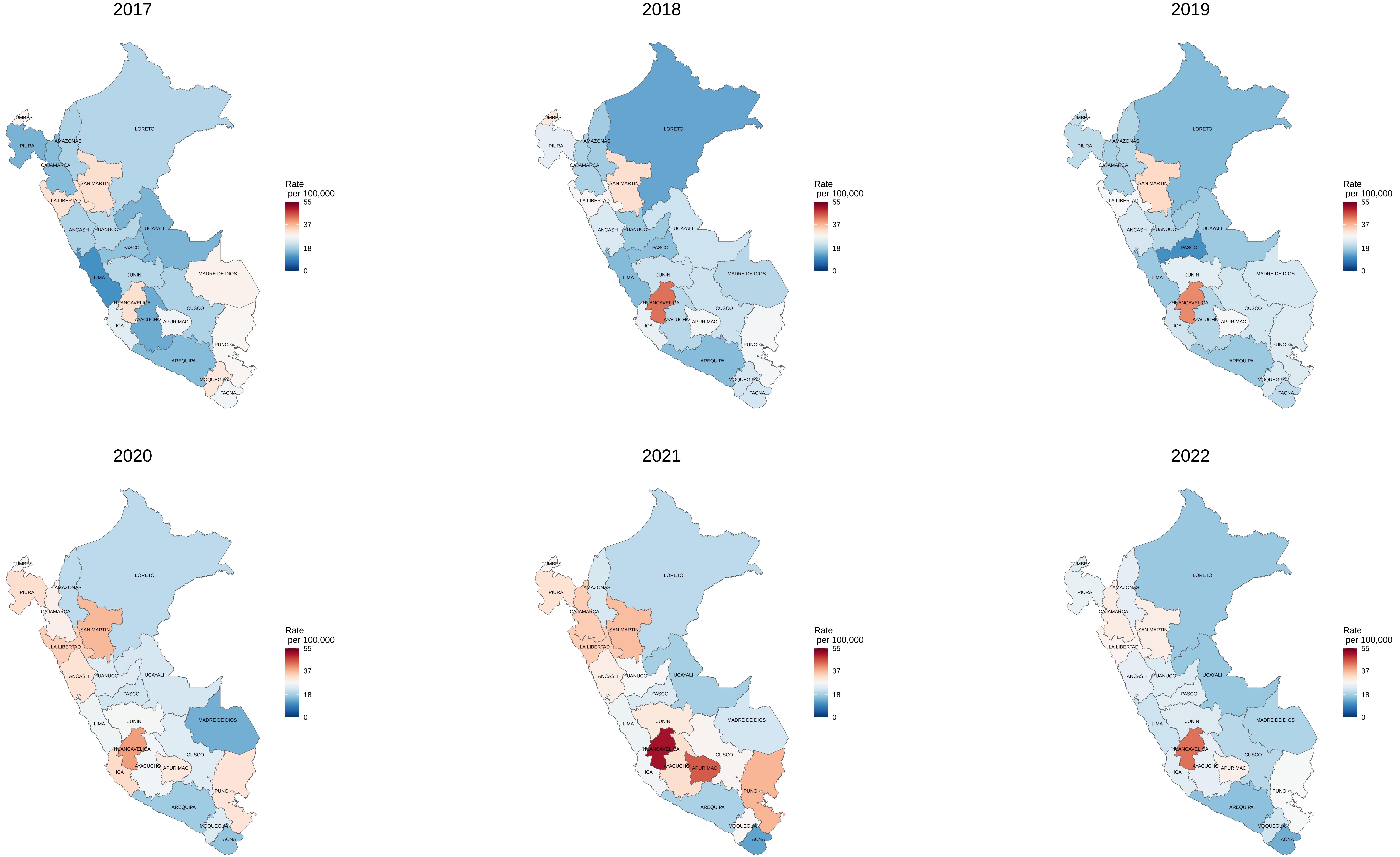

Supplement: Supplementary Fig. 5 — Geographic and temporal profiles of deaths caused by cerebrovascular diseases in Peru between 2017 and 2022. Colors indicate the age-standardized cardiovascular mortality rates. Lines inside the map indicate the boundaries of the Peruvian regions. [file mmc6.pdf]
